# Exposure to Artificial Light at Night and the Consequences for Flora, Fauna, and Ecosystems

**DOI:** 10.3389/fnins.2020.602796

**Published:** 2020-11-16

**Authors:** Jack Falcón, Alicia Torriglia, Dina Attia, Françoise Viénot, Claude Gronfier, Francine Behar-Cohen, Christophe Martinsons, David Hicks

**Affiliations:** ^1^Laboratoire Biologie des Organismes et Ecosystèmes Aquatiques (BOREA), MNHN, CNRS FRE 2030, SU, IRD 207, UCN, UA, Paris, France; ^2^Centre de Recherche des Cordeliers, INSERM U 1138, Ophtalmopole Hôpital Cochin, Assistance Publique - Hôpitaux de Paris, Université de Paris - SU, Paris, France; ^3^ANSES, French Agency for Food, Environmental and Occupational Health & Safety, Maisons-Alfort, France; ^4^Muséum National d’Histoire Naturelle, Paris, France; ^5^Lyon Neuroscience Research Center (CRNL), Waking Team, Inserm UMRS 1028, CNRS UMR 5292, Université Claude Bernard Lyon 1, Lyon, France; ^6^Centre Scientifique et Technique du Bâtiment, Saint Martin d’Hères, France; ^7^Inserm, CNRS, Institut des Neurosciences Cellulaires et Intégratives, Université de Strasbourg, Strasbourg, France

**Keywords:** artificial-light-at-night, light-emitting-diodes, photoreception, biological clocks, ecosystems, anthropogenic impact

## Abstract

The present review draws together wide-ranging studies performed over the last decades that catalogue the effects of artificial-light-at-night (ALAN) upon living species and their environment. We provide an overview of the tremendous variety of light-detection strategies which have evolved in living organisms - unicellular, plants and animals, covering chloroplasts (plants), and the plethora of ocular and extra-ocular organs (animals). We describe the visual pigments which permit photo-detection, paying attention to their spectral characteristics, which extend from the ultraviolet into infrared. We discuss how organisms use light information in a way crucial for their development, growth and survival: phototropism, phototaxis, photoperiodism, and synchronization of circadian clocks. These aspects are treated in depth, as their perturbation underlies much of the disruptive effects of ALAN. The review goes into detail on circadian networks in living organisms, since these fundamental features are of critical importance in regulating the interface between environment and body. Especially, hormonal synthesis and secretion are often under circadian and circannual control, hence perturbation of the clock will lead to hormonal imbalance. The review addresses how the ubiquitous introduction of light-emitting diode technology may exacerbate, or in some cases reduce, the generalized ever-increasing light pollution. Numerous examples are given of how widespread exposure to ALAN is perturbing many aspects of plant and animal behaviour and survival: foraging, orientation, migration, seasonal reproduction, colonization and more. We examine the potential problems at the level of individual species and populations and extend the debate to the consequences for ecosystems. We stress, through a few examples, the synergistic harmful effects resulting from the impacts of ALAN combined with other anthropogenic pressures, which often impact the neuroendocrine loops in vertebrates. The article concludes by debating how these anthropogenic changes could be mitigated by more reasonable use of available technology – for example by restricting illumination to more essential areas and hours, directing lighting to avoid wasteful radiation and selecting spectral emissions, to reduce impact on circadian clocks. We end by discussing how society should take into account the potentially major consequences that ALAN has on the natural world and the repercussions for ongoing human health and welfare.

## Introduction

**FIGURE 1:**
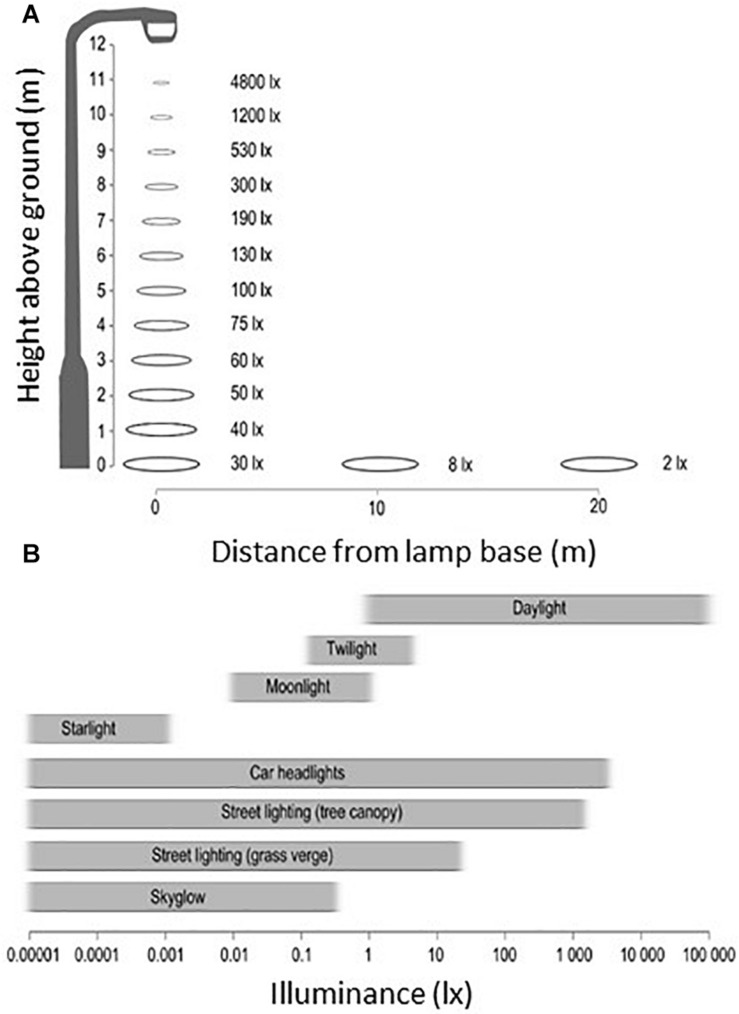
**(A)** Illuminance measured in the horizontal plane from a typical street light (Phillips Cosmopolis, metal halide lamp). The illuminance level decays rapidly with distance to the lamp. **(B)** Comparison of measured illuminance from natural sources of light to artificial light sources – axis is on a logarithmic scale, and bars present approximate ranges based on field measurements. From Bennie et al. (2016). No special permission required.

Human activities are almost exclusively associated with brightly lit environments. The last century has seen an unprecedented increase in the use of Artificial Light at Night (ALAN), with a current ongoing global increase rate of more than 6% per year (Hölker et al., 2010). This is dramatically affecting land as well as aquatic and open sea areas. Mediterranean and temperate zones, mangroves and forest regions in proximity to agricultural areas are particularly affected (Votsi et al., 2017). Today, more than 80% of the worlds population lives under a “lit sky” at night (Falchi et al., 2016), actually affecting up to 99% in Europe and North America and on the increase in the Middle East (Tamir et al., 2017) and Asia (Jiang et al., 2017). ALAN acts both directly and indirectly (through sky glow) upon organisms. The illuminance at ground level can equal that of the full moon (0.01<<1 lx) (Bennie et al., 2015a, 2016; Figure 1) and can even be amplified by the cloud ceiling. ALAN was first intended to detect obstacles, increase road safety and secure potentially dangerous areas at night, but has now been extended to all aspects of human activities, including industrial, commercial, amenity spaces or tourist purposes. Illumination levels often exceed real needs; in some areas the aesthetic aspects (lighting of monuments) or advertising (lighting of commercial areas, shop windows, street signs and illuminated posters) have been given precedent. It follows that untouched natural areas - essential to the development of wildlife - are constantly decreasing. The consequences on biotopes and living organisms (including humans) are multiple. Basic responses and functions related to orientation in space (phototaxis, phototropism) and time (circadian rhythms) are affected by ALAN. These processes are the result of millions of years of evolution, while ALAN-induced changes are operating on a time scale of only a few decades. This is particularly evident when it comes to temporal events, which depend on the predictable alternation of light (L) and darkness (D) during the 24 h LD cycle, day after day and season after season. From the very earliest times of life on earth, organisms developed time-measurement systems - circadian clocks - which allowed them to forecast and anticipate these natural changes, essential for aligning physiological activity with the appropriate time. As a result, most of the basic functions of living organisms are controlled by these internal, genetically determined, clocks. These clocks depend absolutely on the 24 h LD cycle to accurately synchronize their activity with solar time, and in turn they orchestrate a myriad of downstream biochemical, physiological and behavioural events so that the right process occurs at the right time. Thus, changing the natural LD cycle cannot be without consequences for biological organisms. In humans, perturbation of the circadian system results in major physiological impacts (Attia et al., 2019), for example in altered hormonal balance, including melatonin secretion. Melatonin is one key circadian clock output involved in the synchronization of many rhythmic functions; in addition it is suspected to possess powerful anti-oxidative properties (Reiter et al., 1997). In humans, a correlation between ALAN and the appearance of various disorders (activity/sleep rhythms, mental health disorders, energy metabolism, weight gain and obesity, sensitivity to some cancers [breast, prostate]) has been documented quite extensively (Dominoni et al., 2016; Attia et al., 2019) but the level of proof remains low because in most cases the light intensities used are far above the levels encountered in ALAN.

Here, we provide an overview of the tremendous variety of light-detection strategies which have evolved in unicellular organisms, plants and animals. We further give a comprehensive description of the different visual pigments which permit photo-detection in all living organisms from ultraviolet to infrared. The review then moves on to discuss how living organisms actually use light information in a meaningful way, crucial for their development, growth and survival: phototropism, phototaxis, photoperiodism, and synchronization of circadian clocks. These aspects are treated in depth, as their perturbation underlies much of the potentially disruptive effects of ALAN. The review goes into considerable detail on circadian networks in living organisms, since these fundamental features exist in virtually all life forms and are of critical importance in regulating the interface between environment and body. It is necessary to understand the diverse principles underlying their functioning across the different phyla in order to appreciate why ALAN can represent such a disruptive influence. Although much of the data reported in the literature necessarily comes from older lighting technology, the review addresses how the approaching ubiquitous introduction of light-emitting diode (LED) technology may exacerbate, or in some cases reduce, the generalized ever-increasing light pollution. A focus is put on the fundamental role of short wavelength emissions, since these are the most relevant wavelengths when considering signalling through vertebrate photoreceptive tissues and synchronization of central circadian clocks. Nevertheless the paper also stresses that due to the huge range of light detection systems used by living organisms, other wavelengths may also be problematic. Numerous examples are given of how widespread exposure to ALAN is perturbing many aspects of plant and animal behaviour and survival. We examine the potential problems at the level of individual species and populations before extending the debate to the consequences for integrated ecosystems. It also emphasizes additive harmful effects resulting from the impacts of ALAN together with other anthropogenic pressures. The article concludes by debating how these anthropogenic changes could be easily mitigated by more reasonable use of available technology and how society should take into account the potentially major consequences that ALAN has on the natural world and the repercussions for ongoing human health and welfare.

## The Integration of the Light Signal in Living Organisms

*Nothing in biology makes sense except in the light of evolution* (Dobzhansky cited in Lamb, 2013).

The capture of light information goes back to ancestral cyanobacteria, the first known representatives of life on earth, which appeared ∼3.8 billion years ago. It allows organisms to orientate in space (phototropism for animals, phototaxy for plants) and time (synchronization of the endogenous clocks that drive the daily, lunar and annual rhythms of metabolic, physiological and behavioural functions). Living beings have implemented a huge variety of systems and mechanisms in order to capture light, from simple photoreceptive organelles to highly complex structures such as the chloroplast of plants and the camera eyes of vertebrates, insects and cephalopods.

In unicellular organisms, photoreception is mediated by a photoreceptor organelle existing as either a single spot (cyanobacteria, euglena) or a more elaborated structure (dinoflagellates), containing all the elements found in a vertebrate eye, *i.e*., pigment, a cornea-shaped surface, a lens and a lamellar structure (Gehring, 2005, 2011, 2014). It has been hypothesized that these organelles might correspond to chloroplasts incorporated by horizontal transmission, but having lost their photosynthetic activity (Gehring, 2012).

*Cyanophyceae*, the current representatives of the ancestral cyanobacteria are, like the original form, capable of capturing light and ensuring photosynthesis. They exist as single cell units or associated in filaments, and can fix carbon dioxide [CO_2_] and release oxygen [O_2_], but have no chloroplast. Phototaxy and photoperiodic synchronization of circadian clocks have been demonstrated in *Cyanobacteria* (Gehring, 2012), as in the terrestrial *Cyanobacterium Leptolyngbya* sp., which shows two maxima of absorption (λ_*max*_) at 456 and 504 nm. Populations of *Cyanobacteria* are increasing worldwide, favoured by trophic and/or ecological imbalances (including eutrophication of water), and pose major physical (invasion, obstructions) and toxicological (production of dangerous or even deadly toxins) problems (Svrcek and Smith, 2004).

### The Chloroplast of Plants

**FIGURE 2:**
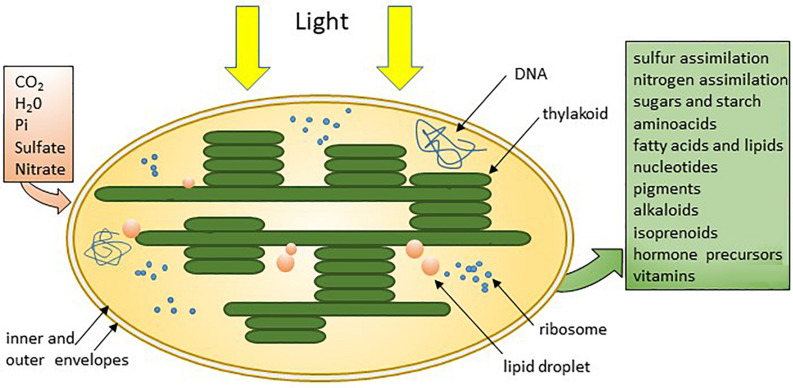
The chloroplast of plants and photosynthetic algae absorbs basic elements and uses sunlight to produce sugar and other organic molecules to fulfil their needs (Kirchhoff, 2019) *@JackFalcón*.

The ingestion of cyanobacteria by primitive eukaryotic cells ∼1.5/1.6 billion years ago led to the formation of chloroplasts (Figure 2), found in the cytoplasm of eukaryotic photosynthetic cells (Kirchhoff, 2019). In the unicellular alga of the *Chlamydomonas* genus, there is one chloroplast per cell, while multicellular plants possess several tens of chloroplasts in one cell, with the leaves showing the highest density. The chloroplast allows photosynthesis, *i.e*., it absorbs light energy to fix inorganic CO_2_ and produces glucose and O_2_ (the highest production of O_2_ is from algae and marine phytoplankton, followed by forests). Moreover, it is involved, by interacting with photoreceptive molecules and circadian clock genes, in the response to light (Jaubert et al., 2017).

### The Photoreceptive Cells and Organs of Animals

**FIGURE 3:**
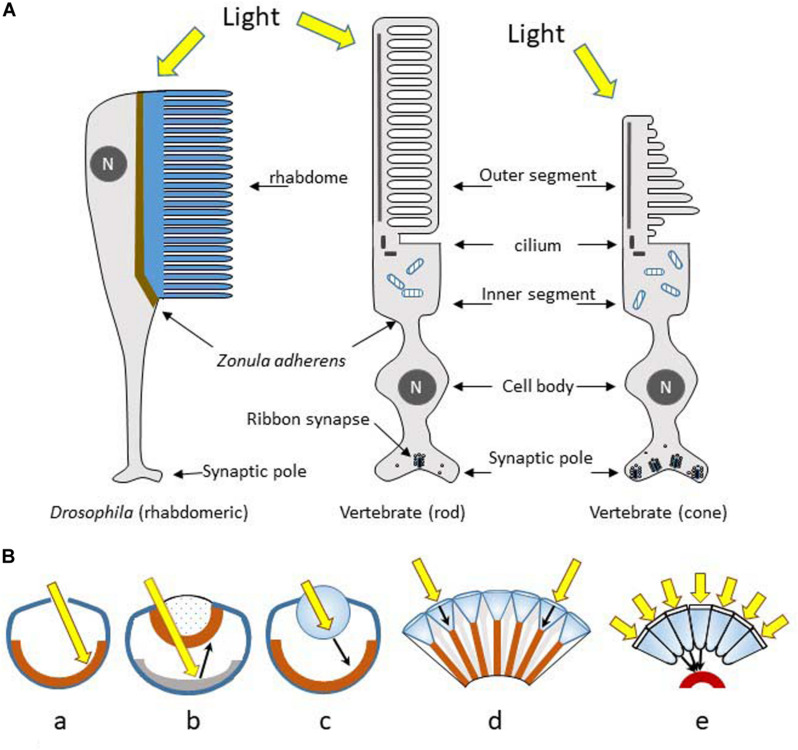
**(A)** Rhabdomeric microvilli-based (invertebrates) and cilia-based (vertebrates) photoreceptors display conserved cell polarity and topology. They evolved most probably from a common ancestor in early Bilateria. The photosensory pole is made of stacks of plasma membrane separated from the baso-lateral membrane by a *zonula adherens*. N, nucleus. **(B)** The main optical designs of eyes: (a) The pinhole eye; light (yellow arrow) falls directly upon the photoreceptors (brown layer). (b) The concave-mirror eye; light crosses the retina, and is then focused back onto the retina upon reflection from a hemispheric reflective mirror (*tapetum*, grey zone). (c) The camera type eye; light is focused by the lens to form an image on the retina. (d and e) The compound eyes; light reaches the photoreceptors exclusively from the small corneal lens (d type) located directly above, or focused through a large number of corneal facets and cones to be directed towards single rhabdoms (e type). Redrawn from Warrant (2019).

The rhabdomeric and ciliary photoreceptors are the two main types of photoreceptive cells found in the animal kingdom. Both show a highly segmented and polarized organization, with a photoreceptive pole made of folds or stacks of membrane, a cell body and an opposing pole for neurotransmission (Figure 3A). Evolution of photoreceptor cells and organs runs in parallel, and studies have shown that eyes and other photoreceptive structures have a monophyletic origin that started with a single prototype (Fain et al., 2010; Gehring, 2012; Lamb, 2013; Gavelis et al., 2015). Evolution led to the appearance of a variety of complex ocular types (Figure 3B). Thus, while the camera-type eye containing ciliary photoreceptors characterizes the eyes of humans and other vertebrates, camera-type eyes are also found in jellyfish and cephalopods, which instead possess rhabdomeric photoreceptors as is the case in most invertebrates. However, coexistence of rhabdomeric and ciliary photoreceptors is not uncommon, as observed in the cephalochordate *Amphioxus*, the living proxy of all vertebrates (Zhang Q. L. et al., 2019). The retina of the hagfish eye, as well as the pineal gland of fish, frogs and sauropsids, is composed mainly of photoreceptor cells connected directly to ganglion cells. The first are of the ciliary type and the second are derived from rhabdomeric photoreceptors, as shown at least in the hagfish (Autrum et al., 2012; Lamb et al., 2007; Lamb, 2013). The retina of all other vertebrates has become more complex, with the appearance of bipolar, horizontal and amacrine cells in an intermediate position. The most recent data indicate that bipolar cells are derived from ciliary type photoreceptors, while the ganglion cells derive from the rhabdomeric line; amacrine and horizontal cells would also belong to the rhabdomeric line (Lamb, 2013).

### Compound and Camera Type Eyes

A dozen different eye structures have been identified in animals, which developed through different evolutionary pathways (divergent, parallel, or convergent) (Shubin et al., 2009). Some are just scattered photoreceptors (alone or a few together) all along the body, found in small invertebrates and in larvae of insects and worms. They are designated as *primitive eyes* because they are associated with a pigmented cell positioned on one side, permitting the perception of light directionality. These structures are simple dosimeters of the surrounding light intensity allowing negative or positive phototaxy (escape or attractive behaviour respectively). In tubular worms these groups of cells form wells or *pit* eyes; the pit eye forms a small hollow in which photoreceptor cells display different orientations, thus allowing spatial detection of light (Figure 3Ba). From these pit eyes appeared the *spherical* concave mirror *eyes* with a pupil, but without a crystalline lens, as seen bordering the mantle of the bivalves (clams, scallops) (Figure 3Bb). More elaborated camera eyes are found in vertebrates, molluscs (squid, octopus), jellyfish, some annelids, arthropods (including spiders), insect larvae and copepods (Figure 3Bc). Finally, the *compound eye*, the most widespread model, is characteristic of insects (75% of existing animal species), most crustaceans, myriapods, some bivalves and polychaetes (Figure 3Bd,e). Compound eyes are formed of identical units called ommatidia, which each contains a cluster of photoreceptor cells surrounded by supporting cells and pigmented cells. Each ommatidium possesses a cornea and a conical lens that focuses light towards the rhabdomeric photoreceptors. In the majority of diurnal species, each ommatidium is isolated from its neighbours by a pigment layer, which makes communication between them impossible (Figure 3Bd). In nocturnal species the absence of pigment allows the diffusion of light from one ommatidium to its close neighbours, conferring a gain of sensitivity (Figure 3Be).

The eye with its retina is not the only structure that allows light detection, as both invertebrates and vertebrates possess additional extra-retinal light sensitive structures.

### Extraretinal Photoreception in Vertebrates

**FIGURE 4:**
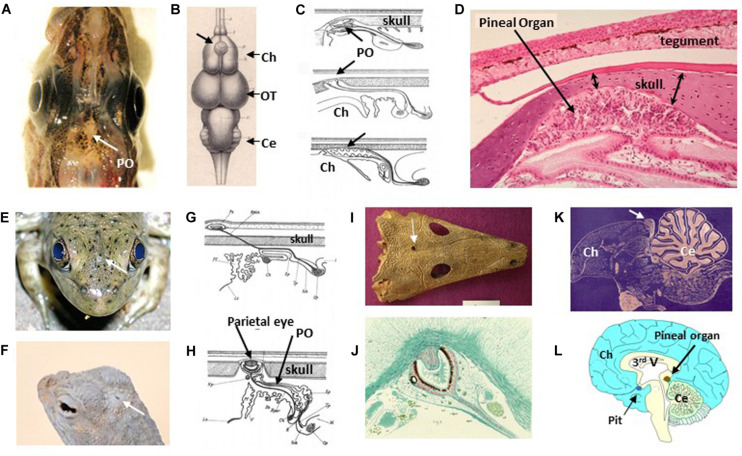
Extraretinal photoreception in vertebrates. (**A**) Dorsal view of the head of the Polar Cod *Boreogadus saida*; the pineal organ (PO) is located in the sagittal axis just behind the eyes in an area with unpigmented meninges (*@JackFalcón*). (**B**) Dorsal view of the brains of the Red Mullet *Mullus surmulletus* showing the location of the pineal organ (thick arrow), located in between the two cerebral hemispheres (Ch); OT, optic tectum; Cer, cerebellum; from Baudelot (1883) (no permission required). (**C**) Schematic sagittal sections through the epithalamus area of, from top to bottom, lampreys, chondrichtyens and teleost fish; from Studnicka (1905). Note that the skull above the pineal organ is thinner, as also seen in panel **(D)** (no permission required). The histological sagittal section is from the Sea Bream *Sparus aurata*; the pineal is located in a kind of large pit below the skull (note that the tegument above also appears thinner) (gift from Professor J.A. Muñoz Cueto, Cadiz, Spain). (**E,F**) Head dorsal views showing the spot position of the frontal organ in the American Bullfrog *Rana catesbeiana* (**E**) and the parietal eye of the Zebra-tailed Lizard *Callisaurus draconoides* (**F**) (arrows) (*@JackFalcón*). (**G,H**) Schematic sagittal sections through the epithalamus areas of frogs (**G**) and lizards (**H**); the pineal organs are located below the skull, while the frontal/parietal eyes are located in the skin connected to the brain by a stalk (Studnicka, 1905) (no permission required). **(I**) Dorsal fossil skull of the ancestral amphibian *Thoosuchus jakovlevi* showing the location of the frontal organ hole just equidistant from the eyes (with permission from https://commons.wikimedia.org/wiki/File:Thoosuchus_jakovlevi.JPG). (**J**) The pineal eye of the tuatara *Sphenodon punctatus* resembles a simplified retina with an eye cup and a lens-like structure; sagittal section from Dendy (1911) (no permission required). (**K**) In the avian brain the pineal organ form a gland in between the cerebral hemispheres and the cerebellum (gift from Professor J.P. Collin). (**L**) In humans the gland is located deep in the brain (*@JackFalcón*).

Aquatic vertebrates, amphibians and lizards possess a pineal complex formed by a pineal gland associated with either a parapineal organ or a parietal eye (depending on the species) (Collin et al., 1988; Falcón, 1999; Figures 4A-J). The gland appears as an evagination of the roof of the diencephalon, located at the surface of the brain. In the majority of cases (particularly in poikilothermic species) the skull directly above the pineal gland is thinner and translucent and the skin is less pigmented (Figures 4A-D). In large fish (*e.g*., the tuna) where the brain is located deep inside the head, a translucent cartilaginous tube directs light from the surface to the pineal gland (personal observations). All these anatomical characteristics allow better light penetration. In addition to the pineal gland, frogs and lizards possess a parietal eye (Figures 4E-J) located between the skull and the skin, which sends a nerve that crosses the skull to reach the brain. In addition, the parietal eye of lizards possesses a lens (Figure 4J). In birds, snakes and mammals these specializations have regressed: the pineal gland of adult mammals is often located more deeply in the brain and has lost its ability to detect light directly, even though they still express the proteins necessary for phototransduction (Figures 4K,L). Furthermore, during development mammalian pinealocytes display morphological features characteristic of ciliary photoreceptor cells but which subsequently regress (Blackshaw and Snyder, 1997).

The pineal epithelium of non-mammalian vertebrates displays the characteristics of a simplified retina as it contains cone-type photoreceptors connected to ganglion cells, the latter sending their axons towards specific brain centres. It is of interest to note that retinal and pineal brain projections overlap in some areas, thus providing convergent light information (Ekström and Meissl, 2003). In contrast to the retina, the pineal organ is only a dosimeter of light intensity, albeit of great sensitivity. In addition to this nervous information pineal photoreceptors also produce the “*time-keeping hormone*” melatonin (see Localization of the Circadian System – Vertebrates) (Falcón, 1999). In the course of evolution snakes and mammals have lost the parapineal and parietal organs, as well as the direct photosensitivity of the pineal gland, and they no longer produce nervous information (Collin et al., 1988). In these species, the pineal cells (pinealocytes), receive light information *via* the retina and a complex nerve pathway; only the nocturnal production of melatonin persists (Klein et al., 1997). Birds display features characteristic of both early and late vertebrates.

In addition to these organized photoreceptive organs, *intracerebral photoreceptors*, the existence of which had been postulated early in the last century (Von Frisch, 1911; Benoit and Assenmacher, 1954), have been found in fish, lizards and birds (Hang et al., 2016; Haas et al., 2017) (see also below Figure 11). Their role remains enigmatic; some may contribute to the annual control of reproduction (Benoit and Assenmacher, 1954).

Finally, ectothermic vertebrates (fish, amphibians, and lizards) possess photosensitive cells on the surface of their skin, which participate in the control of migration in lampreys (Binder and McDonald, 2008), the aggregation/dispersion of skin pigments in fish and frogs (Moriya et al., 1996; Chen et al., 2014), or basking in reptiles (Tosini and Avery, 1996).

### Extra-Retinal Photosensitivity in Invertebrates

**FIGURE 5:**
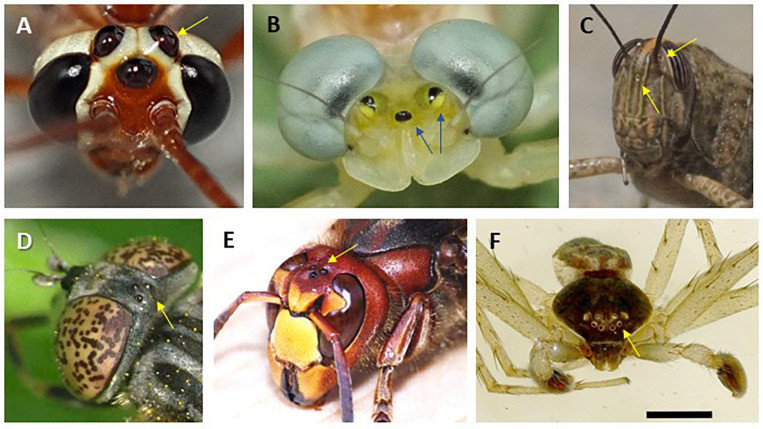
Extra-ocular light perception in various insect species **(A-E)** and eyes of a spider **(F)**. Arrows point to ocellar structures as found in *Netelia sp.* **(A)**, *Heptagenia sp.* **(B)**, grasshopper *Locusta migratoria* **(C)**, *Eristalinus sepulchralis* **(D)**, *Vespa cabro* **(E)**, and *Philodromus dispar* **(F)**. Photo credits: P. Falatico (**A,B,D,E**; @ http://aramel.free.fr/), J Falcón **(C)**, D. Vaudoré (**F**; @https://www.galerie-insecte.org/galerie/ref-183890.htm). No special permissions required.

In addition to their rhabdomeric eyes, insects possess ocelli and eyelets, which may have various shapes and locations (Figures 5A-E). The ocelli of insects are simple lens eyes consisting of a single, large aperture lens, followed by several hundreds of rhabdomeric photoreceptors which converge onto a few tens of interneurons (Berry et al., 2011). *Drosophila* eyelets contain 4 to 6 rhabdomeric photoreceptors and are derived from the larvae visual organs (Helfrich-Förster et al., 2002). Compound eyes and ocelli have a common ancestral origin (Friedrich, 2006), and these extra-retinal photoreceptors are likely to be involved in behaviour and synchronization of endogenous rhythms. Spiders do not have ocelli, but may possess from 1 to 4 pairs of eyes with different functions (Figure 5F)

### Photopigments and Visual Perception

#### Phytochromes

**FIGURE 6:**
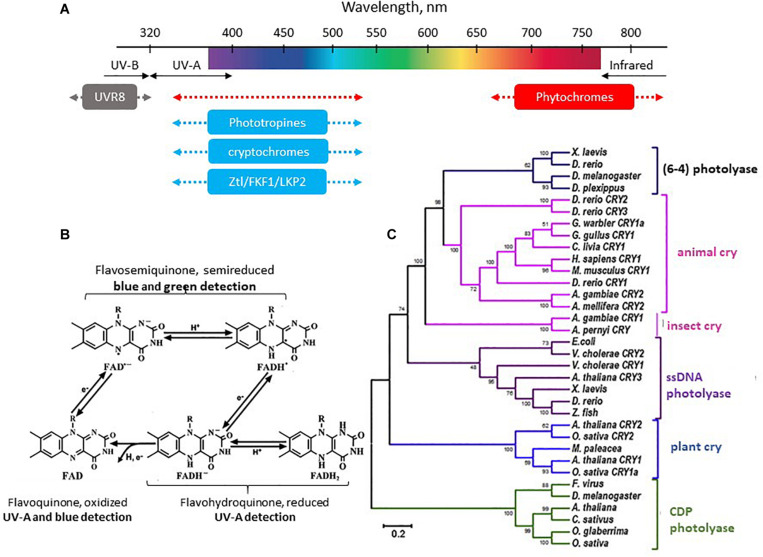
**(A)** The spectral sensitivity of plants. See text and (Huche-Thelier et al., 2016) for details. (**B**) Different states of the flavoquinone cofactor of Cry and corresponding photosensitivity (see text for details). **(C)** Phylogenetic tree of the photolyase/cryptochrome family. Modified from Du et al. (2014), with permission.

Phytochromes are found in plants, fungi, bacteria and cyanobacteria, unicellular algae and diatoms. They are covalently associated with a phytochromobilin as chromophore in plants and cyanobacteria, and biliverdin in other bacteria and fungi (Bhoo et al., 2001; Glukhova et al., 2014; Huche-Thelier et al., 2016). In plants, several forms of phytochromes may be present simultaneously (five in *Arabidopsis thaliana*, three in sorghum, black cottonwood and rice, and two in pea) (Demotes-Mainard et al., 2016). They display maximal sensitivity in the red range of wavelengths, although response to other wavelengths is also observed but with much lower sensitivity (Figure 6A). Phytochromes exists in two states: the inactive state has a sensitivity maximum in the red (580 < λ_*max*_ < 660), while the active state displays its maximum in the infrared (690 < λ_*max*_ < 720). The final effects on downstream regulated processes in the plant depend on the red/infrared ratio (Bhoo et al., 2001; Demotes-Mainard et al., 2016). Light induces bilin photoisomerization and triggers photoconversion from the red to infrared form, prompting activation of the phytochrome HIS-kinase activity and downstream cascades. Darkness induces the opposite and thus the plant needs a dark phase to regenerate the phytochrome from the infrared to red form. Consequently, a natural LD 24 h cycle is essential for the proper synchronization and regulation of physiological cycles in plants (see below).

It is of interest to note that phytochromes also contribute to blue light-dependent regulation either redundantly or synergistically with cryptochromes (Cry; the blue light photoreceptors), and that physical interactions between Cry and phytochromes proteins have been demonstrated (Demotes-Mainard et al., 2016).

#### Cryptochromes

Cry are found in all living organisms (Chaves et al., 2011; Yu and Fischer, 2018). They belong to the photolyase family of proteins and use flavin adenine dinucleotide (FAD) as a cofactor (Figures 6B,C). Photolyases and Cry from the DASH (for *Drosophila, Arabidopsis, Synechocystis*, Human) family (Cry-DASH) are involved in DNA repair (Tagua et al., 2015), which operates between 350 and 530 nm. In plants and animals Cry1 and Cry2 have lost the DNA repairing property. UV-A (λmax 370 nm) and blue (λmax 450 nm) radiations activate an electron transfer and reduction of FAD (initially in an oxidized form) (Huche-Thelier et al., 2016; Liu et al., 2016; Figure 6C). In the animal kingdom Cry are also part of the circadian clock molecular machinery, *i.e*., they ensure both the capture of the light signal (input to the clock) and the function of the clock itself. However, this is not the case in vertebrates where they are no longer light sensitive (see section “Orientation in Time: The Circadian Clocks” below).

As mentioned above, Cry interact with phytochromes in plants, where they also regulate phototropin expression (see section “LOV (Light, Oxygen, or Voltage) Domain Proteins”). They are also involved in the mechanisms of orientation (insects) and magnetoreception (plants, insects, birds) (Chaves et al., 2011; Gehring, 2012). For example, strong magnetic fields reduce plant growth in blue light but not in red light. In Cry deficient (*Cry*−/−) *Drosophila* (*Drosophila melanogaster*) and cockroaches (*Periplaneta americana*), magnetic field orientation function is lost while it is restored in transgenic animals expressing the human gene (*Cry2*+/+) (Bazalova et al., 2016). Similarly, magnetic field orientation through retinal Cry has been demonstrated in migratory birds (particularly nocturnal migrants) and, under dim light intensity, orientation remains correct only at wavelengths under 530 nm (Mouritsen et al., 2004a,b; Solov’yov et al., 2010; Niessner et al., 2011; Fusani et al., 2014).

#### LOV (Light, Oxygen, or Voltage) Domain Proteins

Light, oxygen, or voltage domain containing proteins are a family of blue light receptor proteins that include phototropins, ZTL/FKF1/LKP2 and aureochromes (Suetsugu and Wada, 2013). Phototropins are specific to green plants (land plants and green algae) and ZTL/FKF1/LKP2 to land plants. Aureochromes are specific to photosynthetic stramenopiles, including yellow-green algae (*Xanthophyceae*), brown algae (*Phaeophyceae*), and diatoms (*Bacillariophyceae*).

Phototropins are serine/threonine kinase proteins, which are sensitive to blue and UV-A light (Figure 6A). They use mono-nucleotide flavin (FMN) as chromophore. Studies in *A. thaliana* have demonstrated that phototropin expression is regulated by phytochromes and Cry (Huche-Thelier et al., 2016). Phototropins are involved in the control of phototropic responses (hypocotyl and stem bending, and leaf positioning), the accumulation of chloroplasts and opening of the stomata (responsible for gaseous exchanges between the plant and its environment) (Huche-Thelier et al., 2016).

Like the phototropins, ZTL (Zeitlupe), FKF1 (Flavin-binding Kelch), and LKP2 (LOV Kelch Protein-2) are also associated with FMN and responsive to blue and UV-A wavelengths (Figure 6A; Suetsugu and Wada, 2013). ZTL regulates the circadian clock either directly (through degradation of key clock proteins) but also can indirectly affect the flowering time. LKP2 and FKF1 predominantly control photoperiodic flowering (scent emission, corolla opening, and movements), the former through regulating the circadian clock, and the latter acting downstream of the clock; studies also suggest they contribute to controlling hypocotyl growth (Imaizumi et al., 2003; Dodd et al., 2015; Yon et al., 2016). In fungi, the blue photoreceptor proteins White Collar-1 (WC1) and Vivid (VVD), two LOV domain-containing photoreceptors, are part of the circadian clock machinery (Hurley et al., 2015; Yu and Fischer, 2018; Saini C. et al., 2019).

#### Opsins

**FIGURE 7:**
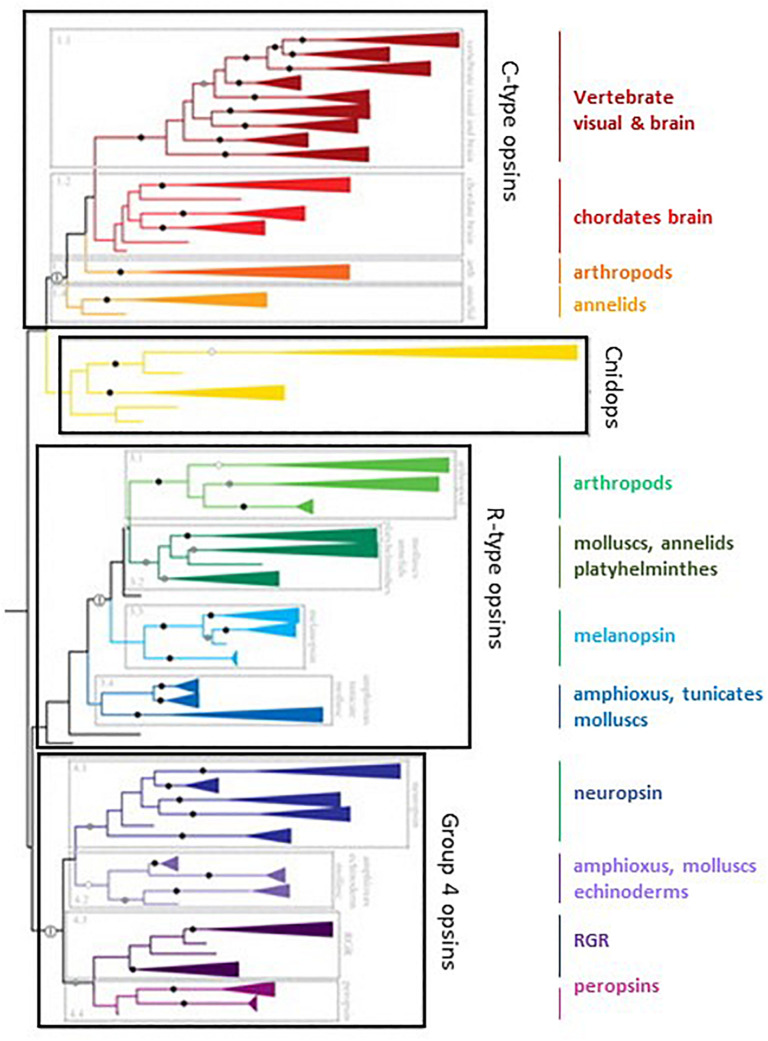
The family of opsins in the tree of evolution. C-opsin family includes the vertebrates visual and brain opsins (Rh1, Rh2, SWS1, SWS2, M/LWS, pinopsins, parapinopsins, vertebrate ancient and parietal opsins), the chordates’ brain opsins (teleost multiple tissue opsins (TMTs), encephalopsins and uncharacterized amphioxus and urchin opsins), the arthropod opsins (honeybee ptersopsin, and uncharacterized insect and *Daphnia pulex* opsins), and the annelids group (uncharacterized *Platynereis* brain and urchin opsins). Cnidops family includes ctenophore and *cnidiarian* opsins. R-type opsins include the arthropod visual pigments (M, LWS, and SWS), the annelid, Platyhelminthes and mollusc visual pigments, the melanopsins (vertebrates’ melanopsin 1 and 2, and amphioxus sequences) and uncharacterized tunicate, amphioxus and mollusc opsins. Group 4 Opsins include neuropsins (four separate clades), amphioxus, sea urchin and scallop opsins, RGR (uncharacterized mollusc opsins) and peropsins (amphioxus and hemichordate opsins). See text and (Porter et al., 2012) for more details. Modified from Porter et al. (2012). No special permission required.

Opsins are members of the G-protein-coupled 7 transmembrane domain receptor (GPCR) superfamily that are associated with the chromophore retinal. This feature is a fundamental distinction between opsins and phytochromes, Cry and LOV-domain containing proteins, which are cytosolic. Upon illumination, retinal isomerizes from the 11-*cis* to all-*trans* configuration (in vertebrates), or all-*trans* to 13-*cis* (in bacteriorhodopsin), triggering the cellular response to light (Shichida and Matsuyama, 2009). Opsins, evolved from a common ancestral molecule ∼ 700 million years ago (Figure 7), show enormous diversity in structure, tissue distribution and function (Porter et al., 2012); more than 1000 sequences are available (Shichida and Matsuyama, 2009). The two categories, microbial (type I) and animal (type II) opsins, share a common architecture but with little sequence homology and have different functions (Kandori, 2015).

##### Type I or microbial rhodopsins

Microbial opsins display great diversity and heterogeneity, comprising archaeal light-activated ion pumps, sensory rhodopsins and halorhodopsins (in bacteria, fungi, cyanobacteria, and dinoflagellates), and rhodopsin channel in green algae. Type I rhodopsins are usually proton or chloride ion (Cl^–^) pumps with green (560 < λ < 590 nm) or blue (λmax: 490 nm) absorption maxima, the latter being particularly observed in deep-sea bacteria (Shichida and Matsuyama, 2009).

##### Type II or animal rhodopsins

Originally opsins were classified in two groups, the C-opsins and the R-opsins, based on the belief they were specific for ciliary photoreceptors (for the former), and rhabdomeric photoreceptors (for the latter). This was shown recently to be an oversimplification (Leung and Montell, 2017). Several animal opsin subfamilies are now recognized, classified as a function of the G-protein they are coupled to and the different intracellular pathways they activate (Porter et al., 2012; Oakley and Speiser, 2015; Terakita et al., 2015). These include the vertebrate visual and non-visual opsins (Gt-coupled), encephalopsin (opn3, Gi/Go-coupled), invertebrate opsin (Go-coupled), cnidarian opsin (Gs-coupled), neuropsin (opn5, Gi-coupled) and melanopsin (Gq-coupled). The function of the two others, peropsin and photoisomerase, is less well known. Type II rhodopsins share less than 20% identity between them. In each group there are some involved in light capture and others whose functions remain unknown. It is noteworthy that the melatonin receptor line appeared after the very first duplication of the ancestral opsin gene (Feuda et al., 2012; Figure 7).

Vertebrate opsins, encephalopsins, Go and Gs opsins are expressed in ciliary photoreceptor cells of the retina and pineal gland of vertebrates, while Gq opsins are expressed in rhabdomeric photoreceptor cells of invertebrates (Shichida and Matsuyama, 2009). In vertebrates, opsins are also expressed in the inner layers of the retina, as is the case for VA (vertebrate ancient) opsin in the inner nuclear layer of non-mammalian vertebrates, or melanopsin in a specific set of intrinsically photosensitive retinal ganglion cells (ipRGCs) in mammals (Jiang et al., 2018) (see also “*Type II or animal rhodopsins*”). Mammals possess a single melanopsin gene (Opn4m, for mammalian), whereas all other vertebrates have at least two (Opn4m and Opn4x [for *Xenopus*]). Chicken Opn4m is restricted to a subset of RGC while Opn4x is found in a different subset of RGC as well as horizontal cells (Verra et al., 2011). There are also long and short isoforms of both Opn4m and Opn4x, which also have differential distributions. In addition to the retina and pineal complex of non-mammalian vertebrates, non-visual light sensitive opsins are also expressed in several brain regions (Hang et al., 2016), scattered throughout the brain (fish) or restricted to the diencephalon (frogs, reptiles and birds) (Pérez et al., 2019). These opsins mediate non-visual light detection regulating many functions, including early development, locomotor activity, or annual control of reproduction, as suspected from very early studies in fish (Von Frisch, 1911) and birds (Benoit, 1935), and now unequivocally demonstrated (Nakane et al., 2010, 2013; Fernandes et al., 2012; Hang et al., 2014, 2016; Currie et al., 2016) (see also Figure 11). Melanopsin (humans) and encephalopsin (rat) have also been detected in the mammalian brain (Nissilä et al., 2012a,b) but it is unknown whether they are linked to a direct sensitivity to light reported for the mammalian brain (Leung and Montell, 2017). A few studies also report the localization of opsins in the brain of a variety of invertebrates (larvae and adult) (Spaethe and Briscoe, 2005; Shiga and Numata, 2007; Donohue et al., 2018). In most of these cases this non-visual photoreception controls behaviour and daily rhythms.

Opsins have also been detected in the skin dermatophores and photophores of vertebrates and invertebrates (Tosini and Avery, 1996; Binder and McDonald, 2008; Pankey et al., 2010; Chen et al., 2014; Baker et al., 2015; Delroisse et al., 2018). These dermatophores participate in the control of pigment aggregation (fish, amphibians), positive (lizard), or negative (gastropod) phototaxis, and the migratory cycle (lamprey). In mice, OPN5 mediates photo-entrainment of clock genes in skin cells (Buhr et al., 2019), and OPN3 mediates blue-light activation of lipolysis in adipocytes (Nayak et al., 2020). Finally, in mammals melanopsin is expressed in blood vessels and iris muscle, being involved in the control of photo-relaxation and pupillary constriction respectively (Leung and Montell, 2017).

##### Wavelength discrimination of opsins

**FIGURE 8:**
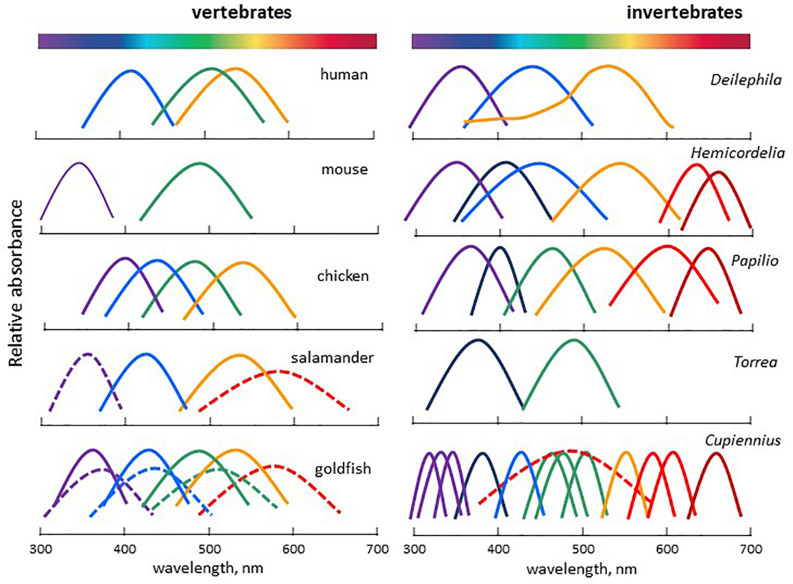
Spectral sensitivity curves of selected vertebrate and invertebrate representatives, illustrating the wide variety of light detection systems encountered. **Vertebrates:** human *Homo sapiens*, mouse *Mus musculus*, chicken *Gallus domesticus*, Salamander *Salamandra*, goldfish *Carassius auratus*. **Invertebrates:** elephant hawk moth *Deilephila elpenor*, dragonfly *Hemicordulia tau*, butterfly Papil*io xuthus*, annelid worm Torr*ea candida*, nocturnal spider *Cupiennius salei*. Adapted and modified from Imamoto and Shichida (2014), Warrant (2019).

Evolution has led to a diversification of opsin genes, resulting from a succession of mutations and whole genome duplications, followed by gains of function or losses of one paralog. The spectral sensitivity peaks of opsins range from ∼310 to ∼ 700 nm in the animal kingdom (between ∼400 and ∼650 nm in vertebrates) (Rowe, 2002; Figure 8). It is not the purpose to discuss here the ways animals discriminate colours; this has been extensively reviewed elsewhere (Lamb, 2013; Olsson et al., 2017; Jacobs, 2018). Rather, we want to emphasize the wide variety of situations - from a single opsin up to several dozens - that can be found from one species to another.

**FIGURE 9:**
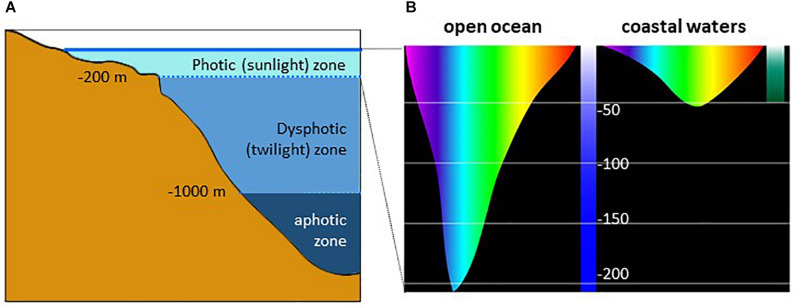
**(A)** Penetration of light into the water column and **(B)** illustration of the depth at which different colours of light penetrate ocean waters. (**B** is modified from the NOAA Office of Ocean Exploration and Research, with permission).

In vertebrate rods, rhodopsin (Rh1) is responsible for the achromatic response (though amphibians and geckos are capable of colour discrimination under scotopic conditions due to two sub-populations of rods detecting light of different wavelengths). The chromatic response is provided by multiple cone sub-types, each expressing one type of opsin, although co-expression of different opsins in one single cone is not an exception (Isayama et al., 2014). Up to four groups of opsins are expressed in cones, maximally sensitive in the UV/blue (SWS1, SWS2), the green/yellow (Rh2) and the red (LWS) ranges (Jacobs, 2018). Whereas most mammals have only two cone pigments (SWS1 or SWS2, and Rh2), diurnal old-world primates have three (SWS2, Rh2, and LWS) (Rowe, 2002; Imamoto and Shichida, 2014). Many marine mammals and a few nocturnal rodents, carnivores, and primates have secondarily lost the S cone pigment and became monochromatic (Figure 8). Invertebrates often display higher diversity as they may possess from a few up to several dozens of visual opsin genes, depending on the species, covering from the UV to the far red wavelengths (Jacobs, 2018; Warrant, 2019; Figure 8). In both vertebrate and invertebrate eyes, photoreceptors and photopigments often display a non-uniform distribution within the retina, in a stochastic/regionalized, regionalized, or ordered manner, providing specific adaptations to the ecological niche they occupy (Viets et al., 2016; Marshall, 2017; Stöckl and Kelber, 2019; Warrant, 2019). Specific adaptation to the local environment is often observed underwater where the composition of the available light depends on many factors, including depth, time of day and other physical parameters (Figure 9). To compensate for these changes, underwater animals have developed mechanisms that alter spectral sensitivity (Temple et al., 2008), including gain or loss of a photoreceptor class, changes in chromophore type [retinal (A1) or 3,4-dehydroretinal (A2)] and expression of different opsin classes or subtypes within a photoreceptor class. The changes may occur during development or depending on the species requirements in adulthood. Light-induced shifts in cone frequency and opsin expression occur in many aquatic species; the expression of opsins is modified by the population habitat and lighting conditions in the Bluefin Killifish, *Lucania goodie*, and during development in Coho Salmon, *Oncorhynchus kisutch*, in a manner that maximizes photonic capture (Fuller and Claricoates, 2011). Similarly, ontogenetic and sexual variations in the expression of opsins have also been described in insects (Temple et al., 2008; Arikawa et al., 2017; Lichtenstein et al., 2018).

## Orientation in Space: Phototaxis, Phototropism

Orientation in space, defined as phototaxis in animals and phototropism in plants, are movements in response to the lighting environment. Positive and negative phototaxis (*i.e*., towards or away from the light stimulus) is most often triggered by blue light detection, but not only (Randel and Jekely, 2016). It may cover the whole spectrum, from UV/A up to near-infrared (Cyanobacteria, Chau et al., 2017; Wilde and Mullineaux, 2017) or just part of it (UV to green in the fruit fly *Drosophila melanogaster* larvae, Humberg and Sprecher, 2017); UV/blue in Hemiptera *Diaphorina citri* (Paris et al., 2017); near-infrared in the zebrafish *Danio rerio* larvae (Hartmann et al., 2018); and green in the bat *Pipistrellus nathusii* (Voigt et al., 2017). Animals (particularly aquatic larvae) may change their preferences during development.

Phototropism characterizes plants and fungi, which, as sedentary organisms, have evolved the ability to alter their growth to optimize light capture and photosynthesis (Goyal et al., 2013; Fankhauser and Christie, 2015; Schumacher, 2017). In most plants and fungi phototropism is triggered by both red and UV-A/blue light, while in flowering plants blue light is the predominant signal. In *Botrytis cinerea*, a pathogenic fungus of plants, light stimulates germination of the conidia, while dark stimulates its growth. Also, germ tube growth is reduced by near-UV, blue and far-red light, which induce negative phototropism, while red light promotes germ tube elongation and induces positive phototropism (Schumacher, 2017). In fact, negative phototropism induced by near-UV/blue light increases pathogenicity, whereas positive phototropism induced by red light suppresses it.

## Orientation in Time: The Circadian Clocks

**FIGURE 10:**
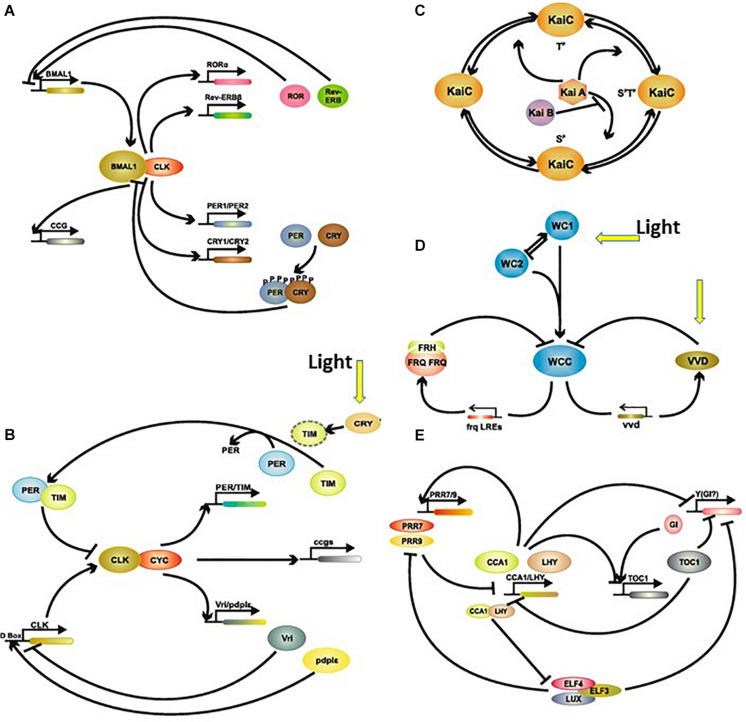
Simplified schematic representation of the circadian clock in (A) mammals, (B) insects, (C) Cyanobacteria, (D) fungi, and (E) plants. For details see Saini R. et al. (2019). Abbreviations: CCA1, circadian clock associated 1; CCG, clock controlled genes; Clk, clock; CRY, cryptochrome; CYC, cycle; ELF, early flowering; FRH, FRQ-interacting RNA, helicase; FRQ, frequency; GI, gigantea; LHY, late elongated hypocotyl; LUX, lux arrhythmo; PER, period; Rev-Erbβ (orphan nuclear receptor family 1); PRR, pseudo-response regulator; RORα, retinoic acid receptor (RAR)-related orphan receptors; TIM, timeless; TOC1, timing of cab expression 1; VVD, vivid; WC, white collar; WCC, white collar complex. Modified from Saini R. et al. (2019) No special permission required.

Orientation in time is provided by the so-called circadian system. This system is made of circadian clocks, which function autonomously and rhythmically with a period of approximately 24 h (Bell-Pedersen et al., 2005). Circadian clocks are present in virtually all living organisms, including cyanobacteria, micro-green algae, plants, fungi and animals (Figure 10). The alternation of light and dark during the 24 h LD cycle is the main environmental input signal to the clocks (although there are others such as food intake, temperature or social interaction), synchronizing and entraining their autonomous activity with the natural world. In return, the clocks produce a number of rhythmic messages, either through direct gene regulation (so-called clock-controlled genes or ccg) or indirectly through activating second messenger cascades. Together, the rhythmic input to the clocks, the clocks themselves and their rhythmic outputs, constitute *the circadian system*. Such an organization governs myriad metabolic, physiological and behavioural processes, thereby synchronizing their activities with the natural periodicities (Reiter, 1991; Falcón et al., 2007b, 2010; Bloch et al., 2013; Table 1). It has been estimated that between 10 and 20% of the genome shows a circadian expression (about 3,000 genes in humans), while a recent study of non-human primates showed that >80% of *de novo* transcripts were rhythmic (possibly under circadian control but also possibly evoked by the light-dark cycle or the sleep-wake cycle) (Mure et al., 2018).

**TABLE 1:** Some examples of demonstrated impacts of the clocks on organisms.

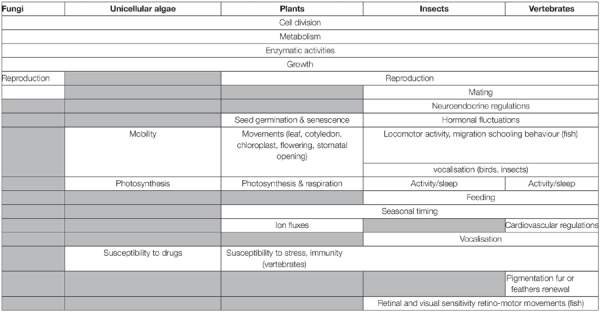

It is believed that circadian clocks appeared very early in evolution as an adaptive function linked to DNA replication. By limiting DNA replication to the night phase, UV-induced damage to DNA could be blocked (Pegoraro and Tauber, 2011). Over geological time selective pressure turned this simple passive process into an active one, allowing anticipation of predictable changes. Among the myriad daily and annual functions displaying clock-controlled rhythmicity are the rest/activity cycle, food intake, flowering, vertical and horizontal migration, growth, reproduction, and many more (Table 1). In addition to their ubiquitous character and the persistence of rhythmic activity under constant light (LL) or darkness (DD) (free-running), other characteristics of a circadian clock include (1) genetic determination (*i.e*., each species has its proper period close to 24 h, but inter-individual variations are observable within the same species), (2) synchronization by other factors (*e.g*., rainfalls, moon cycles, food intake, tides) in addition to the LD cycle; (3) temperature compensation, *i.e*., the clock’s period is not affected by temperature; (4) lengthening or shortening of the period with light intensity under constant light (LL); (5) induction of phase advances or phase delays by light sequences applied at different times under DD; (6) resynchronization by an environmental stimulus once constant conditions have ended. Virtually all cells possess internal clock machinery.

It is worth mentioning that in addition to the circadian clocks many organisms have developed circannual time measuring systems. As is the case for the circadian clocks, circannual clocks are ancestral, ubiquitous, autonomous, entrained by photoperiod and temperature compensated (Lincoln, 2019). The location and mechanisms of the circannual clocks, still poorly understood, are discussed elsewhere (Numata et al., 2015; West and Wood, 2018; Wood and Loudon, 2018; Murphy, 2019).

### Localization of the Circadian System

#### Plants

There is evidence that multiple and distinct circadian clocks are present in different tissues of plants. The first example was obtained from bean plants, in which stomatal opening, photosynthesis, and leaflet movement rhythms displayed different periods under free-running conditions. In addition, it seems that in some cells the 24 h LD cycle is the dominant synchronizing factor, while in others it is the 24 h temperature cycle. The question has arisen as to whether there is a central pacemaker or a hierarchical coupling between different clocks in plants as is the case in animals, and how these different clock activities synchronize with each other. It has been hypothesized that the oscillations in sugar concentrations and/or microRNA (miRNA) might play this role (Endo, 2016).

More is known in invertebrates and vertebrates, where all cells possess molecular clock machinery, forming a network of more or less potent and hierarchically organized units (Falcón et al., 2007b; Dibner et al., 2010; Vatine et al., 2011; Ito and Tomioka, 2016). The hierarchical order varies according to the class and species considered.

#### Vertebrates

**FIGURE 11:**
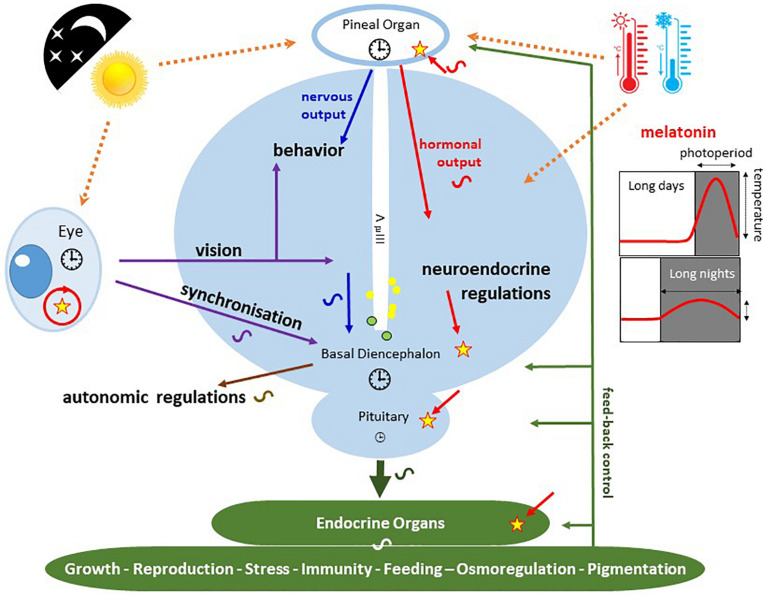
Schematic representation of the photoneuroendocrine organization in the non-mammalian brain. The drawing pictures a frontal section of the brain diencephalic area. Light information is captured by the lateral eyes and the pineal organ. Photosensitive units, expressing different types of opsins, have also been identified along the 3rd ventricle (3rd V; yellow and green circles). Major circadian clock machineries 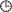 are present in the pineal and retinal photoreceptors as well as in the basal diencephalon (preoptic area [POA] and suprachiasmatic nuclei [SCN]) of lizards and birds. The pineal gland of fish and lizards also integrates temperature information from the external environment. The concomitant action of light, temperature and other internal factors, shapes the rhythmic nervous (blue) and hormonal (red; melatonin) outputs (see text for details), providing a temporal message transmitted to the neuroendocrine axis and downstream targets (peripheral endocrine organs). Melatonin acts through specific receptors (stars) distributed in different tissues and organs. While the main retinal output subserves visual function, a few other fibres also terminate in different parts of the basal diencephalon, where some converge with fibres originating from the pineal gland. Some of the targeted areas also express melatonin receptors. This double or triple input contributes to synchronizing the neuronal activity of the basal diencephalon. In sauropsids the POA and SCN neurons also relay retinal information to the pineal gland. The entire neuroendocrine axis is targeted by ALAN together with multiple other disruptors including temperature rises and pollutants [*e.g*., endocrine disruptors] acting directly or indirectly at different levels of the loop.

In fish and lizards, the circadian system is made of a network of independent and interconnected light-sensitive oscillatory units located in the retina, the pineal gland and probably also in the brain (Tosini et al., 2001; Falcón et al., 2007b). Studies in the zebrafish indicated that virtually all cells from any tissue are light sensitive circadian oscillators (Steindal and Whitmore, 2019), but the great variety of fish species precludes making any generalization. In any case, the pineal gland appears to act as a potent master oscillator, depending on the species (Underwood, 1989; Whitmore et al., 1998; Figure 11). The photoreceptor cells in the retina and pineal gland actually constitute full circadian systems by themselves, as they possess the light transduction machinery that provides input to the clock, as well as the machinery that produces the output signal of this clock, *i.e*., melatonin (Pickard and Tang, 1994; Bolliet et al., 1997; Gothilf et al., 1999). A major difference between the retina and pineal gland lies in the fact that retinal melatonin is generally used and metabolized locally (Figure 11). In the pineal gland, melatonin is typically produced in higher amounts at night than during the day, and is immediately released into the blood or cerebrospinal fluid. The duration of this nocturnal signal reflects the duration of the night, while its amplitude varies with temperature in a species-specific manner (Underwood, 1989; Falcón, 1999). Thus, daily and annual variations in the melatonin secretion profile provide a reliable indication of daily and calendar time, which is used as a time-keeping signal to synchronize physiological and behavioural processes with daily and annual variations in photoperiod and temperature (see section “Clock Outputs and Photoperiodism”).

The strength and reliability of the melatonin time-keeping signal is reflected in its conservation throughout vertebrate evolution. However the modality of melatonin production has been profoundly modified from fish to mammals as a result of dramatic structural and functional modifications of the whole circadian network. In mammals, the circadian components are located in distinct specialized areas. A “master clock” is located in the suprachiasmatic nuclei (SCN; ∼5,000 to 30,000 cells) of the hypothalamus, which interacts with a network of peripheral oscillators (Harder and Oster, 2020). Photoperiodic input to the SCN comes from the retina *via* the retino-hypothalamic tract: while light information encoded by the retina is mostly directed to the visual cortex through ganglion cells (RGC), a small number of these - the melanopsin-containing or intrinsically photosensitive (ip) RGC (see section “Type II or Animal Rhodopsins”) - send information to the SCN (as well as numerous other brain nuclei) (Do, 2019). One downstream effector of the SCN is the pineal gland, with its rhythmic melatonin production; but the gland has lost all intrinsic photoreceptive and circadian properties (Collin et al., 1988; Klein et al., 1997). Rhythmic information from the SCN is transmitted to the pineal gland *via* a poly-synaptic neural pathway (Klein et al., 1997; Falcón et al., 2007b). The few studies performed in Sauropsida (birds and reptiles) indicate that melatonin secretion by the pineal gland is controlled by both direct and indirect photosensitivity (Cassone, 2014).

#### Invertebrates

Insects include more than 1 million species, displaying a huge diversity in all aspects of organization and life style, and there is much variation in the anatomical organization of the circadian network in the insect brain (Bloch et al., 2013). Despite this diversity, there are striking similarities in the principal organization of circadian clocks. In the fruit fly *Drosophila melanogaster* the network consists of a few hundred neurons (Hermann-Luibl and Helfrich-Foerster, 2015). A master clock is located in scattered nuclei located in the optic lobes and brain, composing a neuronal network (Tomioka and Matsumoto, 2010; Hermann et al., 2013; Hermann-Luibl and Helfrich-Foerster, 2015). These neurons utilize mainly neuropeptides as signalling molecules, including pigment-dispersing factor (PDF), which appears to be well-conserved in putative master clock neurons of all insects studied so far (including apterygotes, orthopteroids, coleoptera, hymenoptera, lepidoptera and diptera Tomioka and Matsumoto, 2010). In *D. melanogaster*, PDF is considered as the main output factor of clocks, acting as a neuromodulator and synchronizing signal between the different central clock neuron clusters (Helfrich-Forster et al., 2011; Hermann et al., 2013). In addition to these central clocks, there is evidence indicating that many other organs or tissues, either nervous (eye and eye stalk, antenna) or peripheral (gustatory system, Malpighian tubules, prothoracic gland, epidermis secreting endocuticle, testis and germinal vesicle), express circadian clock properties (Tomioka et al., 2012). Photoperiodic information captured by the ocular, and in some instances the ocelli photoreceptors, entrains the central oscillators, which in turn deliver information to slave peripheral oscillators. In crickets and cockroaches this pathway is essential (Tomioka and Matsumoto, 2010; Tomioka et al., 2012). In other species (*e.g*., *Drosophila*) the central brain and some of the peripheral oscillators are fully integrated circadian systems as they are able to capture light and thus synchronize their clocks and output functions *in vitro* (Tomioka et al., 2012), in a manner similar to that described for the zebrafish (Whitmore et al., 1998). In the eye, the Rh1 and Rh6 rhodopsins are implicated in entrainment to red light (*D. melanogaster*), while in the brain and peripheral oscillators it is likely to be the UV A/blue pigment Cry1 (drosophila *D. melanogaster* and Monarch butterfly *Danaus plexippus*) (see section “Phytochromes”) (Tomioka and Matsumoto, 2010). It is noteworthy that the central brain circadian system is highly plastic as photoperiodic changes have been reported in fibre distribution or number of clock neurons (Shiga, 2013).

### The Molecular Mechanisms of Circadian Clocks

The purpose here is to highlight the universality of the underlying principle as well as the wide range of situations encountered regarding the qualitative aspects of clock entrainment by light (Bhadra et al., 2017; Saini R. et al., 2019).

Irrespective of the organism studied, the molecular clock mechanism consists of one or more transcription/translation negative feedback loops of varying complexity (Figure 10). Because the functioning of the clock involves similar operating mechanisms with different molecular actors, it is thought that clocks have appeared independently several times during evolution (Pegoraro and Tauber, 2011). The number of these actors varies from a few (fungi, green algae) to many (plants, animals) (Saini R. et al., 2019). The molecular mechanisms of the circadian clocks, have been described in detail in Cyanobacteria, fungi (*Neurospora crassa*), plants (*Arabidopsis thalliana*), green algae (*Chlamydomonas reinhardtii*, *Ostreococcus tauri*), insects (*Drosophila melanogaster*) and several representatives of vertebrates including human (Tomioka and Matsumoto, 2010, 2015; Ukai and Ueda, 2010; Nakamichi, 2011; Peschel and Helfrich-Forster, 2011; Vatine et al., 2011; Hurley et al., 2015; Ito and Tomioka, 2016; Koritala and Lee, 2017; Gil and Park, 2019). Strong conservation of the operating modes is observed between insects and mammals, including at the level of the molecular actors (Tomioka and Matsumoto, 2015; Figure 10). It is worth mentioning that post-transcriptional regulation and protein modification, such as phosphorylation and oxidation, have been hypothesized as alternatives ways to building a ticking clock (Millius et al., 2019).

### Light Input to the Clock

Light is the main input to the clocks. The effects on the circadian timing systems depend on the intensity, duration, spectrum and pattern of the light stimulus; for a review in humans see Prayag et al. (2019). In the animals investigated thus far, short and middle wavelengths are strongly involved in synchronization and entrainment. In vertebrates, the effective wavelengths are comprised between 420 and 500 nm, the highest efficiency being obtained between 450 and 480 nm (Ramos et al., 2014; Prayag et al., 2019). In mammals, this corresponds to the spectral response of melanopsin from the ipRGC of the retina (see “*Type II or animal rhodopsins*”). However, it is not excluded that the mechanisms of light-induced clock entrainment are more complex than believed. Indeed, it has been observed that colour opponent mechanisms can induce phase advances or phase delays in the circadian rhythm, depending on light intensity and spectral composition, in the pineal organ of fish, frogs and lizards (Spitschan et al., 2017). Opposing effects of wavelengths on circadian phase shifts have been shown in the cave-dwelling bat *Hipposideros speoris* (blue *vs*. green) and wild rabbit *Oryctolagus cuniculus* (blue *vs*. yellow). It is noteworthy that a subset of ipRGC, sensitive to UV is also indirectly sensitive (*via* cone perception) to yellow wavelengths in the mouse *Mus musculus*.

In insects such as *D. melanogaster* and other flies, Cry1 is involved both in light capture (see section “Cryptochromes”) and molecular function of the clock (Figure 10; Saunders, 2012). Cry1 is sensitive to blue light (λ_*max*_ 470). In addition, Rh1 and Rh6 are implicated in entrainment to red light, and Rh1, Rh5, and Rh6 to green and yellow light (Tomioka and Matsumoto, 2010).

In plants, a variety of situations is observed regarding the wavelengths that entrain the clocks. In terrestrial higher plants, *e.g*., *A. thaliana*, phytochromes (see section “Phytochromes”) mediate the effects of red and infrared wavelengths (λ: 700-750 nm), while Cry1 and Cry2 mediate the effects of blue light (Figure 10; Chen et al., 2004; McClung, 2006). In microalgae such as *C. reinhardtii* the clock is reset by a wide range of wavelengths: violet, blue/green and red (Niwa et al., 2013; Ryo et al., 2016). Finally, in fungi the light entrainment of the clock is mediated by the WC1 blue photoreceptor species (Bhadra et al., 2017).

### Clock Outputs and Photoperiodism

Clocks control a wide range of peripheral oscillators and related downstream processes, many of them vital, to keep in phase the myriad rhythmic events that take place over the course of a day or a year. We present below a short overview (summarized in Table 1), with the help of a few examples taken from unicellular organisms, fungi, plants and animals.

#### Unicellular Algae, Plants, and Fungi

*Neurospora crassa* was the first fungi in which endogenous circadian control of its sexual and asexual daily rhythms of reproduction was demonstrated (Zámborszky et al., 2014; Hurley et al., 2015). The asexual cycle consists in the production of conidia during the subjective night, and similar rhythms in conidiospore formation have now been reported in Myxomycetes, Zygomycetes and Ascomycetes (Correa and Bell-Pedersen, 2002). In *N. crassa* and other multinucleated fungi (*Physarum polycephalum* and *Aspergillus nidulansone*), LD cycles also synchronize the timing of mitotic cycles (Edmunds, 1988; Hong et al., 2014). The involvement of the circadian clock has been demonstrated in *Neurospora*, in which 15-20% of the genes are clock-controlled (Zámborszky et al., 2014) (Table 1).

Virtually all functions of unicellular algae are rhythmic and synchronized by the LD cycle, including metabolism, enzymatic activities, photosynthesis, cell division cycle, mobility, morphology and chromosome topology, and even the susceptibility to drug treatments or infection by viruses (Table 1; Edmunds, 1984). The outputs are generated by 24 h LD rhythms in gene transcription/translation (Welkie et al., 2019).

Similarly, in more distantly related plants such as *A. thaliana*, the rhythms controlled by the circadian clock are plethoric, including gene expression, Ca^2+^ fluxes, chloroplast movements, stomata opening, flowering, cotyledon and leaf movements, metabolic and hormonal activities, or defence against pathogens (Barak et al., 2000; Table 1). In a large scale study comparing nine representatives of Archaeplastida, including unicellular algae (*Cyanophora paradoxa, Porphyridium purpureum, Chlamidomonas Reinhardtii*), pluricellular algae (*Klebsormidium nitens*), mosses (*Physcomitrella patens*), early vascular plants (*Selaginella moellendorffii*), and late vascular plants (*Picea abies, Oryza sativa, A. thaliana*), it was found that they had similar diurnal transcriptional programs, despite large phylogenetic distances and dramatic differences in morphology and lifestyle (Ferrari et al., 2019; Table 1).

#### Animals

##### Vertebrates

The circadian clocks of vertebrates contribute to controlling a myriad of rhythmic metabolic, physiological and behavioural functions (Boissin and Canguilhem, 1998; Table 1). One main output signal from the circadian system of vertebrates is melatonin, the hormone secreted principally at night by the pineal gland (“Vertebrates” and Figure 11; Collin et al., 1988; Ekström and Meissl, 2003; Falcón et al., 2007a).

At the molecular level, the clocks govern rhythmic variations in plasma levels of ions, carbohydrates and lipids, and of brain and plasma steroids, and monoamines (serotonin, dopamine) (Delahunty et al., 1980; Olcese et al., 1981; Takahashi, 1996; Tong et al., 2013; Mendoza and Challet, 2014; Hernandez-Perez et al., 2015; Vancura et al., 2016; Song et al., 2017); furthermore, it also regulates the expression of genes or activities of enzymes involved in these changes (Falcón, 1999). At the physiological level, the neuroendocrine system, from the hypothalamus to the pituitary gland and peripheral organs, displays daily and annual fluctuations, which contributes to controlling a wide range of functions as critical as growth, reproduction, stress response, food intake, immunity or osmoregulation (Falcón et al., 2010; Tonsfeldt and Chappell, 2012; Wood and Loudon, 2014; Challet, 2015; Kim et al., 2015; Leliavski et al., 2015; Figure 11). The cardiovascular system (blood pressure and heart rate) and neuronal electrical activity (electroretinogram and electroencephalogram) do not escape the rule as they also fluctuate rhythmically (Boissin and Canguilhem, 1998; Peters and Cassone, 2005; Cameron and Lucas, 2009; Talathi et al., 2009; Wood and Loudon, 2014; Petsakou et al., 2015; Cavey et al., 2016; Paul et al., 2016; Figure 11 and Table 1). Finally, in many tissues, clocks also control the cell division cycle (Boissin and Canguilhem, 1998; Steindal and Whitmore, 2019), as well as some adaptive cellular movements including retino-motor movements (the respective elongation and retraction of cones and rods observed in fish and amphibians retinas at the L-to-D and D-to-L transitions) (Kwan et al., 1996; Song et al., 2017). Accordingly, dozens of behavioural activities display daily and annual rhythms, including locomotor activity and sleep, schooling behaviour (fish), pigmentation or fur renewal, vertical (fish) and horizontal (all vertebrates) migration, behavioural thermoregulation (fish), vocalization (fish, birds), food intake, mating and reproduction, etc…(Zachmann et al., 1992; Lincoln et al., 2006; Cancho-Candela et al., 2007; Kantermann et al., 2007; Foster and Roenneberg, 2008; Kulczykowska et al., 2010; Cassone, 2014; Ruf and Geiser, 2015; Table 1).

##### Invertebrates

The data on invertebrates are not as abundant as for vertebrates, and relate mostly to insects, although more and more studies refer to marine invertebrates. All indicate that the clocks mediate the effects of photoperiod and temperature on a myriad of rhythmic daily and seasonal events (Helfrich-Forster et al., 2011; Arboleda et al., 2019). The most obvious relate to feeding (*e.*g., foraging in bees, and moths, bugs and mosquitoes bites), reproduction (*e.g*., courtship behaviour, mating and reproduction), and growth (larval and adult development, diapause, longevity) (Helfrich-Forster et al., 2011; Bloch et al., 2013; Rougvie and O’Connor, 2013; Table 1).

The neuromodulator PDF, important for transmitting clock information to downstream effectors, also acts as a circulating hormone (Bloch et al., 2013). There is anatomical and physiological evidence that the invertebrate circadian system influences circulating levels of endocrine signals, including juvenile hormone (JH), ecdysteroids, and “pheromone biosynthesis activating neuropeptide.” JH plays key roles in regulating the reproductive physiology and behaviour in insects as well as in controlling the age-related division of labour in social insects. The levels of transcripts of JH biosynthetic enzymes in the *corpora allata* display strong daily rhythms in the bee, mosquito and fruit fly. In the haemolymph, the circulating levels of JH, JH-binding protein and JH-degrading enzymes also display strong circadian dependent variations (Bloch et al., 2013). It is believed that the JH oscillations mediate the circadian rhythms in the levels of neurotransmitters (pheromone biosynthesis activating neuropeptide), and hormones (octopamine; serotonin; dopamine) thought to be important for locomotor activity or reproduction (including the production of pheromones, courtship, mating, and gamete production) (Koutroumpa and Jacquin-Joly, 2014). Similarly, it is suspected that PDF controls the rhythmic production of the prothoracicotrophic hormone involved in the regulation of ecdysteroids, which control moulting (Table 1).

Finally, the electrical activity of invertebrates’ eyes (electroretinogram) and of the entire visual system display circadian fluctuations (Hernandez and Fuentes-Pardo, 2001). In the Praying Mantis, *Hierodula patellifera*, rhythms are associated with cyclic changes in the colour of the eyes, neural control of eye movement, and gross locomotor activity (Schirmer et al., 2014).

## Impact of Alan and LEDs on Living Organisms

“*Nature is perfect. I keep a diary. I write on which day of the month the flowers bloom and on which day of the month the insects begin to sing. Year after year, these dates hardly vary. They are very regular, this is one of the laws of nature. What goes with the laws is nature. Nature is in accordance with the laws. That’s why I believe people should live by imitating nature… Nature does the truth in silence*.”

Master Ekiyo Miyazaki (1902 – 2008).

### The Generalization of LED Illumination

Initially motivated by the desire to provide more energy-efficient light sources for public lighting (Nair and Dhoble, 2015), the use of LED now concerns a wide range of technological, socio-economic and commercial applications. A variety of sources contributes directly or indirectly (glowing) to outdoors LED lighting: offices and homes, street lighting (Figure 1), vehicles, traffic signs, commercial advertising, tourism (architectural and landscaping enhancement), industry (factories, greenhouses), or recreational (outdoor and indoor sports) areas. Aquatic environments are also affected (shorelines and coastlines in urban and suburban areas, offshore platforms, commercial routes or fishing areas, especially night fishing). From such considerations it can be argued that investigations on the effects of outdoors LED are closely associated to those of ALAN, a situation clearly unfavourable to the preservation of the night sky.

Artificial lighting in general, and LEDs in particular, add to the list of numerous anthropogenic pressures that, decade after decade, are changing an equilibrium that has resulted from millions of years of evolution, affecting the tree of life, of which man is only one branch among thousands of others. In the vast majority of cases, studies investigating the impacts of a given factor consider mainly the effects on human health, while impacts on the animal and plant kingdoms are considered mainly within the context of improving productivity in order to satisfy growing human needs of livestock and derived products. This egocentric view is currently directing most of the research on LED; furthermore, the majority of studies are conducted in a controlled environment, while the impact on non-domesticated species and ecosystems are rarely taken into account.

We have given above an overview of the incredibly wide range of strategies that have been developed by unicellular and multicellular organisms (i) to capture and transduce light information into messages conveyed to appropriate targets, (ii) to orientate in space and time and ultimately (iii) to accomplish their essential biological needs. The development of internal clocks reflects adaptation to the highly predictable and reliable variations of the photic environment allowing anticipation and harmonization of the myriad of biological functions to the daily and annual changes of photoperiod. It is therefore not surprising that disturbances of this photic environment, whether in quality, quantity or duration, have more or less marked impacts on living organisms. Below we review, through a few representative examples, how human activities and ALAN, alone or in combination with other anthropogenic factors, alter individuals, species and communities.

### Economical Purposes

#### Cultivation of Microorganisms and Plants

Many studies highlight the interest of LEDs for the greenhouse cultivation of plants (Yeh et al., 2014; Nair and Dhoble, 2015; Singh et al., 2015; Dueck et al., 2016; Urrestarazu et al., 2016; Rehman et al., 2017), fungi (Wu et al., 2013; Kim et al., 2014), and unicellular microalgae (Schulze et al., 2014) of agronomic, ornamental or medicinal interest. One major focus resides in the possibility to choose a particular wavelength (of narrow spectral range) or a combination of wavelengths, targeting specific aspects of plant physiology in greenhouse environments (Rehman et al., 2017). In plants, day length, light intensity, and light quality affect morphology, growth and development. The effects of light (whether by LED or other sources) on fungi and plants depend on the range of frequencies they detect. Table 2 summarizes the effects of different frequencies on the metabolism and physiology of plants. For example, far blue and UV lights are useful for eliminating bacterial and viral infections (Yeh et al., 2014; Kumar and Engle, 2016; Kim et al., 2017), while an adequate combination of blue and red/infrared wavelengths provides optimal effects in terms of metabolism (*e.g*., photosynthesis, lipid synthesis, energy production), germination, cell division, budding, growth, flowering, nutritional value and taste, or production of compounds with high added value (ergosterol, carotene). Little information is available on the impact of green lights.

**TABLE 2:** Effects of wavelengths on plants (from Xu et al., 2016).

λ (nm)	Impact
280-315	minimal impact on morphology and physiology
315-400	Weaker chlorophyll absorption, impacts on cyclical activity & growth (tissues & stem)
400-520	Chlorophyll and carotenoid absorption proportion is the largest, the biggest influence on photosynthesis
520-610	Decreased absorption by pigments
610-720	Chlorophyll absorption rate is low, significant effects on photosynthesis and cyclical activity
720-1000	Minimal absorption, effects on photosynthesis, blooming and seed germination
>1000	Convert to heat

However, several factors need careful attention:

(1) The effects of a wavelength or cocktail of wavelengths depend on the species and, within the same species, on sex and stage of development; they also depend on intensity, positioning, periodicity or frequency of exposure (Dueck et al., 2016; Hernandez and Kubota, 2016). For example, cyanobacteria grow preferentially under green, yellow and red light, whereas microalgae preferentially grow under blue (420 < λ < 470 nm) or red (λ = 660 nm) light.
(2) Potentially toxic compounds might be produced. For example, studies on Lamb’s Lettuce (*Valerianella locusta*) indicate the plants can accumulate beneficial (polyphenols) as well as unwanted (nitrates) compounds depending on the proportions of red and blue light used (Dlugosz-Grochowska et al., 2016; Wojciechowska et al., 2016). In contrast, in *Brassica alboglabra* nitrate concentration in shoots increased significantly when grown in the shade compared to lit areas, while it was reduced after red- and blue-LED lighting (He et al., 2019).
(3) The importance of plant and microbiome interactions, rarely taken into account, need more careful investigation, as light can affect both plant physiology and surrounding microbiome density and composition (including pathogenic species) differently (Alsanius et al., 2019).

Thus, while the use of LED in the food industry is promising, it is still at an experimental stage, and studies must be conducted on a case-by-case basis, as the physiological processes involved in the responses to light are incompletely understood (Delabbio, 2015) “*For practice, more research is needed to optimize plant distances, light strategies and light intensities to make the technology more profitable and sustainable*” (Nair and Dhoble, 2015; Moerkens et al., 2016).

#### Breeding

As mentioned above, the quality (λ), quantity (intensity), and duration (photoperiod) of the light phase play a major role in the regulation of metabolism, physiology and behaviour in the animal kingdom (Maisse and Breton, 1996; Malpaux et al., 1996; Falcón et al., 2007b, 2010; Rocha et al., 2013; Espigares et al., 2017). During decades, manipulation of the surrounding light conditions has been part of the protocols used to control food intake, larval development, growth rate and reproduction in farm animals (Delabbio, 2015). For a given lighting condition, the response is species-specific; differences may also exist within the same species as a function of age, sex, or geographical location (Pan et al., 2015).

The use of LEDs to substitute for “conventional” lighting in aquaculture farms, poultry and mammal housing is the subject of an intensive promotional campaign, which emphasizes the advantages provided by LEDs (controlled choice of wavelength and lower running costs) (Delabbio, 2015). Field applications are still scarce (Pan et al., 2015; Yang et al., 2016). Studies aim to compare the effects of LEDs to conventional lighting on growth, food intake and conversion efficiency, weight gain, egg production or behaviour (aggressiveness, exploration) (Huber-Eicher et al., 2013; Pan et al., 2015). In spite of a noticeable increase in the number of publications, the data remain too scarce for definitive conclusions to be drawn. Some examples are reported below.

##### Insects

Light-emitting diodes have been used to select wavelengths that favour reproduction of the Black Soldier fly *Hermetia illucens*, a tropical fly species with great potential for the processing of several types of organic waste and by-products (Oonincx et al., 2016), or for trapping pests like the Cigarette Beetle, *Lasioderma serricorne* (Miyatake et al., 2016) and other harmful species (Cohnstaedt et al., 2008).

##### Corals

A positive impact of LEDs compared to other light sources has been reported on the growth of the ornamental corals *Stylophora pistillata* and *Galaxea fascicularis*, but not of *Acropora formosa* (Wijgerde et al., 2012; Rocha et al., 2013). In *A. Formosa* and *S. pistillata*, wavelength affects macro- and micro-morphology (Rocha et al., 2014).

##### Molluscs

The predatory Dog Whelk *Nucella lapillus* exerts strong top-down control on biodiversity in intertidal coastal regions. Under nocturnal white LED illumination mimicking street lighting (∼22 lx), individuals displayed higher activity, disregarded the presence of other predators, and increased feeding on mussels (Underwood et al., 2017). The effects of LEDs of different wavelengths were also examined in the abalone *Haliotis discus* (Gao et al., 2016). It was found that under blue or green light, the survival and growth rates, food intake, and food conversion efficiency were lower than in groups exposed to red or orange light; the former displayed enhanced anaerobic metabolism and energy loss, while the latter showed higher amylase and cellulose activity.

##### Fish

Several studies reported the impact of different wavelengths on growth, hormonal control of reproduction, stress and pigmentation, biological rhythms (clock gene expression, melatonin secretion), thyroid activity (T3, T4) and expression of opsin genes (Rh, melanopsin) (Jung S. J. et al., 2016; Takahashi et al., 2016). They emphasized the interest and the potential use of white, mono or dichromatic LEDs in aquaculture and breeding, but underline the necessity of rigorous experimentation. Blue LEDs have the potential to kill unwanted pathogens in aquaculture plants. For example, LED light at 405 and 465 nm were efficient in Olive Flounder (*Paralichthys olivaceus*) and Carp (*Cyprinus carpio*) culture farms to eliminate *Miamiensis avidus* and *Edwardsiella piscicida* respectively (Roh et al., 2018). However, at 405 nm the dorsal part of the retina was damaged after 14 days in *P. olivaceus*, outlining the possibility that these treatments might have deleterious side effects on the fish itself. In the fisheries industry, there is evidence that LEDs are being used by fishermen to attract species of interest (Park J. A. et al., 2015; Kehayias et al., 2016).

##### Birds

The use of LEDs in avian farms has increased dramatically in recent years, with the aim to reduce production costs combined with improving reproduction and growth and reducing stress (Huber-Eicher et al., 2013; Parvin et al., 2014a,b; Yang et al., 2018; Arowolo et al., 2019). A huge variety of protocols have been used that take into account age and sex of animals, as well as light quality, intensity, periodicity and duration. For example, red LEDs advance sexual maturation while decreasing aggression compared to green or white LEDs in hens *Gallus domesticus* (Gongruttananun, 2011; Huber-Eicher et al., 2013); the effects were due to quality and not the amount of light provided. Green LEDs promote egg growth, and blue, green or yellow LEDs, used alone or in combination, promote immune defence and improve meat quality (Parvin et al., 2014a,b). The authors stated that more research on these aspects is needed in order to standardize intensities, durations, and exposure wavelength.

### Impact on Species in Their Environment

#### Microorganisms and Plants

Artificial nocturnal illumination with white LED can influence biomass and community composition of terrestrial photoautotrophs^1^. In diatoms and sedimentary *Cyanobacteria* white LED (6300 K) induce quantitative population remodelling, loss of annual variations in population composition, decreased respiratory activity and redistribution of sedimentary microbial populations; these modifications are likely to change the CO_2_ cycle and induce carbon accumulation in sediments (Hölker et al., 2010). Similarly, in freshwater ecosystems, three weeks of exposure to ALAN (white LED, 20 lx) decreased periphyton (the mixture of algae, microbes, cyanobacteria and detritus) biomass and the proportion of Cyanobacteria, while increasing the proportion of Diatoms (Grubisic et al., 2017, 2018a,b). In addition, it was shown that the replacement of high-pressure sodium (HPS) lamps by white LED at intensities commonly found in urban waters (∼20 lx), induced similar but stronger effects (Grubisic et al., 2018b). Autotrophs within periphyton communities form the base of aquatic food webs and as such constitute a fundamental element in aquatic ecosystems. More studies are needed that should include the marine environment in which ALAN disturbs synchronized diel vertical migrations of zooplankton and where the vast majority of the zooplankton pelagic community exhibits a strong light-escape response in the presence of artificial light (Ludvigsen et al., 2018).

In plants, the intensity of lighting used in urban and suburban districts as well as on highways is sufficient to affect their physiology (Bennie et al., 2016; Massetti, 2018). The described effects of night lighting (including by LEDs) include tree leaf colouring, retention/abscission (on deciduous trees), budding, flowering, growth, or defence against pathogens. In the case of fungi involved in litter decomposition of streams, and which play a key role in the carbon and nutrient dynamics of stream ecosystems, ALAN can alter community structure and composition, resulting in inhibition of litter decomposition (Liu et al., 2020).

#### Animals

There is no longer any doubt that ALAN affects phototaxis and circadian rhythms, and consequently any ensuing functions and behaviours. It is beyond the scope of the present review to discuss the impacts of ALAN on human health and related studies (Attia et al., 2019). Rather, we focus on the available data that can aid understanding its impacts in the wild.

##### Invertebrates

One of the major problems with ALAN is the attraction of insect communities by nocturnal lights, and most of the studies on invertebrates focus on this (Honnen et al., 2019). In general, these studies indicate the observed effects depend on the species and quality of light (Longcore et al., 2015; Park J. H. et al., 2015; Silva et al., 2015; Wakefield et al., 2015; Acharya et al., 2016). In Ohio (United States) LED lamps attract a large number of insects, all species combined (Knop et al., 2017), but only half as much as incandescent lamps at an equivalent energy (Justice and Justice, 2016). In the Netherlands the number of Fog Moths (*Operophtera brumata*) caught outdoors was higher in the areas directly lit by LEDs than in the shadow, and the effect depended on the wavelength (in the following order of potency: green > white > red) (Geffen et al., 2015). Inhibition of food intake has also been observed regardless of light wavelength (Van Langevelde et al., 2017). In contrast, foraging activity was increased in spiders (*Eriophora biapicata*) (Willmott et al., 2018). Reproductive success and growth of moths and spiders are also compromised by ALAN: sexual activity of females and attraction of males to females were disrupted by LED lighting of different wavelengths (red > white > green) in *Operophtera brumata* (Geffen et al., 2015). In *E. biapicata*, a 20 lx white LED at night accelerated maturation but reduced the number and size of juveniles (Willmott et al., 2018). In the mosquito *Culex pipiens f. molestus* (familiar in urban areas), ALAN (cool-white LED, 100-300 lx) applied during the first 3 h of the night phase resulted in females producing fewer and smaller eggs (Honnen et al., 2019); in addition, males and females were less active during the ALAN phase but females became more active thereafter. The sex-dependent differences were also seen in clock genes because the same ALAN conditions induced upregulation of *Cycle* in females and down regulation of *Clock* in males, with consequences on the median relative expression of clock genes and activity cycles (Honnen et al., 2019).

In fireflies ALAN has been rated as the second most serious threat after habitat loss, showing adverse effects on populations (Lewis et al., 2020). ALAN interferes with the production and perception of courtship messages, glowing (*e.g*., *Lampyris noctiluca*) or flash dialogues (*Pteroptyx maipo, Photuris pyralis*). Ultimately, such effects impinge upon reproduction of the species (Bird and Parker, 2014; Owens et al., 2020).

In coastal areas of Chile the sandy beach isopod *Tylos spinulosus* is active at night. ALAN (120 lx; white LED) disrupted isopod locomotor activity and circadian rhythms, resulting in a dramatic avoidance of lit areas at night (Duarte et al., 2019).

##### Fish

###### Reproduction

Night lighting affects reproduction of fish in several ways, and in a complex manner (Figure 11). White LEDs of low intensity inhibited gonadotrophin expression (FSH, folliculo-stimulating hormone; LH, luteinizing hormone) in female Perch *Perca fluviatilis*, whereas monochromatic wavelengths (blue, green, or red) had no effect (Brüning et al., 2016). In the same study ALAN of different intensities (0.1 to 100 lx) inhibited secretion of the time-keeping hormone melatonin regardless of the LED wavelength used (Brüning et al., 2016). Under similar conditions melatonin levels were also affected in Roach *Rutilus*, whereas no effect was seen on gonadotrophin expression (Brüning et al., 2018a). However, in field experiments using HPS lamps, abundance of sex steroids (17β-estradiol; 11-ketotestosterone) and FSH and LH mRNA was reduced in both *P. fluviatilis* and *R. rutilus*, while melatonin levels were not significantly affected (Brüning et al., 2018b). In dwarf fish, *Chrysiptera parasema* and *C. cyanea*, nocturnal exposure to monochromatic, but not white, LEDs promoted gonadal maturation (Shin et al., 2013; Yeh et al., 2014), the most effective wavelengths being green and blue in *C. parasema*, and red in *C. cyanea*. Oestradiol production was also stimulated in *C. parasema* (Shin et al., 2013), and gonadotrophins were stimulated in goldfish, *Carassius auratus*, when daytime illumination was replaced by monochromatic LEDs; green light, which also increased the expression of VAL-opsin, was the most potent (Song et al., 2015). White LED light at night (∼23 lx illuminance) totally inhibited hatching in the Clownfish *Amphiprion ocellaris*, although no impact was found on the frequency of spawning or fertilization success (Fobert et al., 2019). The authors speculated that fish with similar spawning strategies might respond similarly to ALAN.

Altogether, it is apparent that ALAN can interfere with components of the reproductive axis in fish (Figure 11). These conclusions are supported by long term laboratory experiments in zebrafish *D. rerio*. After 1 year under LL (fluorescent bulbs, 300 lx) the molecular clock was disrupted in the ovary, oestrogen levels were increased (∼50%) while progesterone levels were decreased (∼25%), and plasma, retina and brain melatonin rhythms were abolished (Khan et al., 2018). More importantly perhaps, there was molecular and histological evidence of tumorigenesis in the ovaries of the ALAN group. ALAN also affected the whole transcriptome, including genes involved in tumorigenesis and other physiological disorders (Khan et al., 2018).

###### Behaviour

**FIGURE 12:**
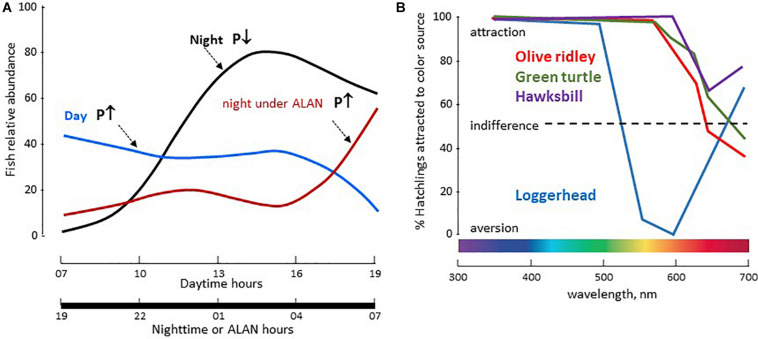
(**A**) Observed abundances in fish populations from the harbour of Sydney (Australia) under a 12L/12D cycle plotted in 15 min bins. Under the natural LD cycle the number of fish is higher during night (black line) than during day (blue line); they were sedentary at night with low predation activity (**P↓**), while displaying a predatory behaviour during day (**P↑**). ALAN (40-50 lx, warm LED light), transformed the nocturnal pattern into a diurnal one. Modified and adapted from Bolton et al. (2017). (**B**) Orientation response of 4 species of sea turtles hatchlings to coloured light sources. Olive Ridley Sea Turtle *Lepidochelys olivacea*, Green Sea Turtle *Chelonia mydas*, Hawksbill Sea Turtle *Eretmochelys imbricata*, were attracted when illuminated with UV-A to yellow wavelengths. The Loggerhead Sea Turtle *Caretta* differed in that UV-A to green lights were attractive, but yellow wavelengths were repulsive, an effect reversed by red illumination. For details see (Witherington and Martin, 2003) from which the figure was modified and adapted.

Behaviour is also affected in coastal and fresh water fish (Figure 12A). In two lakes of Ontario (Canada), locomotor activity, and thus energy expenditure, of Black Bass *Micropterus dolomieu*, which nests and protects its offspring, was abnormally high in the presence of continuous or intermittent night lighting (White LEDs, 40 lx at the water surface) mimicking traffic lights (Foster et al., 2016). Intermittent lighting was the most aggressive. The effects were observed both during day and night phases and rendered offspring survival more random. Parental care occurs in 60% of freshwater fish families; ALAN could thus have negative consequences on many species that build nests in lake and river littoral zones. An escape behaviour has also been reported in the Largemouth Bass, *Micropterus salmoides*, in response to LED lights (green, yellow, orange, and red) pulses applied during the day time (Sullivan et al., 2016). This may be related to the observation that street lighting acted as a light barrier in Atlantic salmon, *Salmo salar*, fry (Riley et al., 2013) (and section “The Migrating Atlantic Salmon - A Case Study”). Light disrupted the daily rhythm in fry dispersion and delayed downstream migration. These changes in migratory behaviour may impact on fish fitness and increase predation risk.

Altogether, the available studies, although scarce, suggest that ALAN is “*an unpredictable threat for light sensitive species, communities, and consequently biodiversity*” (Brüning et al., 2018a,b), a danger potentiated by the observations that responses depend on the species and their life strategies (Fobert et al., 2019).

##### Frogs

Only a few studies explored the physiological consequences of ALAN on amphibians, all indicating it is likely to have negative effects on populations. Thus, white LED lighting (equivalent to that produced by street lighting) affects the nocturnal distribution as well as choice of preferred substrate of the unisexual Blue-Spotted Salamander (*Ambystoma lateral jeffersonianum*), but had no such effect on the frog *Rana sylvaticus* (Feuka et al., 2017). The authors concluded that these choices are likely to affect the survival of both species as salamanders must choose a substrate of lower nutritional quality while frogs become more exposed to nocturnal predators. In field experiments, nocturnal LED light (Blue/green spectrum and intensities consistent with those found under street light) reduced larvae metamorphosis duration and juvenile growth in the American toad *Anaxyrus americanus* (Dananay and Benard, 2018). In addition ALAN also affected periphyton biomass, as mentioned before (section “Breeding”). In the Pennsylvanian wood frog *Lithobates sylvaticus* tadpoles, ALAN (indoors white LED) did not change metamorphosis duration but reduced hatching success (May et al., 2019). Furthermore, while *A. americanus* larvae kept a high rate of activity under illuminated night (comparing to daytime), *L. sylvaticus* tadpoles moved less, and after metamorphosis individuals exposed to ALAN were more susceptible to NaCl challenge and trematodes. Reduced activity and altered metabolism were also reported in male common toads, *Bufo* exposed for 20 days to ALAN (white LED; 0.1, 5, or 20 lx illuminance) (Touzot et al., 2019). As the effects were observed at the onset of the breeding period the authors suggested that ALAN could be a serious threat for many nocturnal amphibian species.

##### Reptiles

Although scarce, studies on reptiles indicate ALAN is a major threat. Many studies focussed on sea turtles of coastal areas all around the world; the impact of ALAN on nesting and hatchlings has been documented since the early 80’s (Witherington and Martin, 2003). Sea turtle nesting and hatching occur at night, generally eggs from one nest hatch together, though sometimes a main group of hatchlings may be preceded or followed by smaller groups (Witherington and Martin, 2003; Robertson et al., 2016). Coastal light at night causes spawning at sea and abandonment of nests or alteration of the choice of nesting site in several species of the Caribbean islands (Green Turtle, *Chelonia mydas;* Hawksbill Turtle, *Eretmochelys imbricate*; Leatherback Turtle, *Dermochelys coriacea*). Modelling studies predict light pollution will substantially accelerate the extinction of these species (Brei et al., 2016). Similar data were obtained along Australian shores: *C. mydas* hatchlings were disoriented in the presence of shore lights, and those that reached and entered the sea returned to shore if reaching an area lit by shore-based artificial lights (Truscott et al., 2017). Low-pressure sodium-vapor (LPS) yellow lights were believed to provide a more “turtle-friendly” environment in Loggerheads and Green Turtles, as UV-blue and green wavelengths were the most attractive to hatchlings, while the red ones were not (Witherington and Martin, 2003; Figure 12B). However, more recent investigations indicated LEDs emitting in the red (narrow band, 600-670 nm, λ_*max*_ 640 nm) and yellow (wide band, 600-750 nm, λ_*max*_ 620 nm) induced total disorientation of Loggerhead hatchlings in their race towards the sea at equal intensities (Robertson et al., 2016). The maximum effect depended on the number of lighting spots with amber coloured emissions being the most potent in the absence of moonlight. According to the authors, coastal lighting is a dramatic threat to the species.

Little information is available concerning terrestrial reptiles, although a long list of species, likely to be affected by ALAN in urban and suburban locations, has been documented (Perry et al., 2008). Recent observations on the nocturnal behaviour and activity patterns of two species of diurnal anole from Antigua (West Indies; Leach’s Anole *Anolis leachii* and Watts’s Anole, *A. wattsi*), describe an increased activity under ALAN, albeit restricted to males and primarily related to the increase in the number of arthropods attracted by light (Maurer et al., 2019).

##### Birds

There is abundance of data on the impact of ALAN on birds with dozens of publications over the last five years (Dominoni et al., 2013a, 2016; Zhao et al., 2014; Ronconi et al., 2015; de Jong et al., 2016b; Krüger et al., 2017; Raap, 2018; Jiang et al., 2020). Overall, ALAN disrupts the circadian system in both sedentary and migratory birds, affecting phototaxis and altering endogenous daily and annual rhythms. These effects are observed both inland and above the sea where lights emitted by drilling and extraction platforms, as well as vessels, have significant effects. Birds are attracted by light and become disoriented. Collisions with solid structures (or contact with flames from chimneys) have dramatic effects, causing the death of hundreds or even thousands of individuals (Ronconi et al., 2015; Krüger et al., 2017; Rodriguez et al., 2017). These effects may vary depending on the quality and intensity of the light source, LPS and LED being less harmful than metal halide lamps (Ronconi et al., 2015). In addition, when collision is avoided, the migratory birds may end up turning in circles around the platforms, negatively impacting the trajectory and migration time, energy expenditure and ultimately survival. In addition to collision, ALAN affects the stopover habitat use by inland migrating birds, which avoid bright areas (McLaren et al., 2018).

Artificial-light-at-night also induces indirect effects through the disorganization of the birds’ circadian system. In a study comparing rural and urban tree sparrows *Passer montanus* of Mizoram (India) differences were found in the phase and/or amplitude of clock gene mRNA abundance in the retina, pineal gland and hypothalamus (Renthlei and Trivedi, 2019). Downstream ccg genes (including melatonin receptors) also differed in their rhythmic expression and abundance between rural and urban birds. In addition, the rhythm in melatonin production itself was also different. The mismatches between the rhythms of different components of *P. montanus* circadian system and effectors seen in urban birds are likely to have consequences on circadian controlled processes. Indeed, indoors experiments with *P. montanus* of the Beijing area (China) have shown that ALAN alters the whole neuroendocrine reproductive axis (Zhang X. J. et al., 2019); mRNA abundance corresponding to FSH, THS (thyroid stimulating hormone) and Dio2 (deiodinase II) were upregulated with low illuminance levels (85 lx; cold white) and down regulated with high illuminance levels (150 and 300 lx) of ALAN. The rise and amount of plasma LH and oestradiol were earlier and higher in the 85 lx group, and later and lower in the other groups, indicating reproduction timing and efficiency were altered.

Light-emitting diodes covering a wide spectrum (450 < λ < 700 nm) affect daily rhythms of locomotor activity, body temperature, singing and sleep (duration and quality), night-time production of melatonin, proliferation of brain stem cells, immunity and oxidative stress markers, as reported in several species, including the Great Tit *Parus major* (Ouyang et al., 2015; Raap et al., 2015, 2016a,c; de Jong et al., 2016a, 2017; Raap, 2018), Blackbird *Turdus merula* (Dominoni et al., 2013b), Indian Weaver Bird *Ploceus philippinus* (Kumar et al., 2018), Japanese Quail *Coturnix japonica*, chicken *G. domesticus* and King Quail *Excalfactoria chinesis* (Saini C. et al., 2019), Zebra Finches *Taeniopygia guttata* (Moaraf et al., 2020), and Weaver *Ploceus philippinus* (Singh et al., 2012). In laboratory experiments the effects were dose-dependent (0.05 to 5 lx) and varied with the spectral composition (de Jong et al., 2016a, 2017). In urban areas with conventional street lighting, whenever possible tits avoided night-time illumination (de Jong et al., 2016b). ALAN did not affect markers of oxidative stress (Casasole et al., 2017), but corticosterone levels were higher in chicks under white, red, blue or green LEDs (8 lx) (Ouyang et al., 2015). The effects depended on wavelength and distance between the nests and light source. The number of chicks was also decreased in nests under ALAN. Finally, a negative correlation was found between the number of chicks and corticosterone levels (Ouyang et al., 2015), as well as the distance to the light source (de Jong et al., 2015). Under similar conditions no effect was observed on the Black Flycatcher (*Ficedula hypoleuca*).

Artificial-light-at-night also has impacts on reproduction, and affects the annual breeding rate (Le Tallec, 2014; Longcore et al., 2015). In the Blackbird *T. merula*, a 0.3 lx white light induced a one-month phase advance in the annual rhythm of reproduction (monitoring size and functionality of testes and steroid levels) and moulting (Dominoni et al., 2013b). Interestingly, these parameters differed depending on whether the blackbirds were captured in the city or forest, suggesting that habitat induced adaptive changes in the species. Similar data were obtained from the California Jay, *Aphelocoma californica*, in which testosterone, oestradiol, melatonin and LH plasma levels showed sex-specific alterations under low night-time (3.2 lx) illumination (*i.e*., corresponding to that measured in suburban areas at Davis, CA, United States) (Schoech et al., 2013). In Mockingbirds *Mimus polyglottos* and American Blackbirds *T. migratorius*, ALAN induced dose-dependent changes in the dawn onset of singing and courtship behaviour as well as the start of the breeding season (Longcore, 2010).

Finally, the impact of continuous or partial nocturnal illumination on avian circadian clocks is believed to be responsible for ametropia (abnormal refractive condition) (Nickla and Totonelly, 2016) and developmental delays observed in the visual system and eye of young birds, as is the case in primates (Attia et al., 2019).

Altogether, it appears that the avian responses to ALAN are complex, depending very much on the species, sex and age, geographical area as well as on the experimental conditions. In general, the data obtained under laboratory conditions agree with those obtained on site, using measures of urban lighting (Raap et al., 2016b).

##### Mammals

The potential influence of ALAN and LEDs on mammals has not been investigated in depth and concerns only a limited number of species, despite the fact that 69% of mammalian species are nocturnal. ALAN affects nocturnal activity in terrestrial vertebrates: an inverse correlation has been found between surfaces lit by ALAN and mammalian species richness (Duffy et al., 2015; Ciach and Frohlich, 2019). Mice (Rotics et al., 2011a,b) and small tropical forest mammals (Bengsen et al., 2010) are less active under ALAN to minimize the risk of predation. The opposite holds true with diurnal and crepuscular species, more active under ALAN, particularly those feeding on insects (Lacoeuilhe et al., 2014; Minnaar et al., 2015; Russ et al., 2015). A study compared the impact of LPS and white LED lighting during the day (equal intensity, but with a stronger blue component for LEDs) in rats (*Rattus norvegicus*): LED-lit individuals had higher nocturnal melatonin levels (seven-fold increase), increased food intake, drinking, growth and lipid levels (in several tissues), while protein levels were lower (Dauchy et al., 2016). In the blood, arterial O_2_ and CO_2_ rhythms were not altered, but titres were higher under LEDs. Conversely, glucose, leptin, lactate and corticosterone levels were decreased in the LED-lit rats, with either a phase delay (leptin) or a phase advance (glucose and lactate) under LEDs compared to LPS lights.

In the normal life cycle of the Siberian hamster (*Phodopus sungorus)*, gonads, body mass, and number of spermatogonia are reduced in winter (short photoperiod), fur becomes thicker and white (Table 1), all changes being adaptations to rigorous winter conditions. Under ALAN (5 lx, white light) these changes were no longer observed; hamsters maintained summer characteristics (long photoperiod) (Ikeno et al., 2014). In addition, a number of genes displayed altered expression, including Per1 (clock function), Mel1a (melatonin receptor), eya3 (involved in development), or TSH, Gonadotrophin Inhibiting Hormone or Gonadotrophin Releasing Hormone (GnRH) (reproduction). Finally, locomotor activity and immune responses were altered, also observed in mice *Mus musculus* exposed to similar conditions (Fonken and Nelson, 2014). Mice also displayed altered body temperature. Changes in body temperature and locomotor activity were also observed in the Gray Mouse Lemur *Microcebus murinus* exposed during 2 weeks to either artificial moonlight (of the same irradiance as natural full moonlight) or to ALAN (HPS street lamps, Le Tallec et al., 2016). The daily rhythm profiles of locomotor activity were altered between the two paradigms in both phase and amplitude, in both males and females, irrespective of the season. Other changes in ALAN-exposed animals included the frequency and duration of torpor phases (decreased), urinary oestradiol (higher in post oestrus and pre-oestrus females), testosterone levels, and testes size (progressively increased in males). Finally, it is worth mentioning that in rats, non-human primates and sheep, disruption induced by ALAN results in major changes in foetal development (shorter pregnancy, low weight), with long-term impacts on offspring at different metabolic and physiological levels (Torres-Farfan et al., 2020).

The most abundant documentation in mammals relates to the family of bats, which account for 30% of existing mammals; 17% of the 1232 bat species are in danger of extinction. Their nocturnal activity is by far the greatest of all known nocturnal mammals. They make short-distance (for foraging and feeding of offspring) and long-distance (search for hibernation sites or at transition sexual/rest phases) trips. Bats show a great wealth and diversity of habitats (caves, cellars, trees, etc.) and eating habits, some being carnivorous (insectivores for the majority) others vegetarian (fruits, flowers, or nectar). They occupy all stages of the food chain, and play a particularly important role in regulating insect populations (including pests), pollination or seed dispersal (Boyles et al., 2011; Kunz et al., 2011). The duration, beginning and end of the nocturnal activity is specific to each species. Thus, lactating females start early at dusk compared to other individuals, while pregnant females or slow-flying species start later at night. Insectivores (*Pipistrellus* spp. and *Nyctalus* spp.) have activity peaks at evening twilight, and it is the presence of prey rather than levels of light that regulates these behaviours (although *Pipistrellus* avoid flying under bright light, Mathews et al., 2015). In contrast, slow fliers (gleaners) or nocturnal butterfly eaters (*e.g., Barbastella barbastellus, Myotis nattereri, M. bechsteinii*) are more sensitive to lighting and prefer complete darkness.

Bats have been classified in two groups depending on their tolerance or intolerance to ALAN (Lacoeuilhe et al., 2014). Field studies indicate that ALAN has a greater impact than land loss (due to urban extension and agriculture) on the distribution of different species of bats (*Pipistrellus pipistrellus, Eptesicus serotinus, P. kuhlii, P. nathusius, Nyctalus leisleri*) (Azam, 2016). In natural and urban environments ALAN (LPS or white LED) affects bat behaviour (Polak et al., 2011; Stone et al., 2012; Lewanzik and Voigt, 2014, 2017; Leliavski et al., 2015; Mathews et al., 2015; Minnaar et al., 2015; Azam, 2016; Rowse et al., 2016). Among the most notable effects are a delay to leave the nest, decreased sexual activity, changes in flight speed and paths (trajectory, height) as well as significant increases in collisions (∼25%) in the presence of lit obstacles (indicating that echolocation is not the only navigation tool for some species). The effects are species dependent. Gleaners or bats relying 100% on echolocation (*Rhinolophus* spp., *Plecotus* spp., *Myotis* spp.) emerge more rarely and modify their routes in a midnight light environment, while large fast-flying insectivorous species (*Lasiurus* spp., *Eptesicus* spp., *Nyctalus* spp., *Pipistrellus* spp.) are attracted by ALAN (Lewanzik and Voigt, 2014; Mathews et al., 2015; Azam, 2016). Others like *Eptesicus bottae* accelerate flight speed and stop hunting insects (Polak et al., 2011).

In Southern England and Wales, population richness and activity of *P. pipistrellus*, *Nyctalus* spp., *P. pygmaeus* and *Myotis* spp. did not change after replacement of LPS by white LED in the street lamps (Rowse et al., 2016). Another investigation found no change in activity of the fast-flying *P. pipistrellus, P. pygmaeus and Nyctalus/Eptesicus* spp. (even at the highest illuminance of 49.8 lx), but observed a significant reduction in activity of slow-flying bats, *Rhinolophus hipposideros* and *Myotis* spp. (even at low light levels of 3.6 lx) (Stone et al., 2012). In another field study close to Nurnberg (Germany) it was found that replacing conventional mercury vapour street lamps with white LEDs changed the impact of ALAN on urban bats: some species showed a clear reduction in their activity (by 45% in *P. pipistrellus*) while others did the opposite (*Myotis* spp.) (Lewanzik and Voigt, 2017). This indicates that replacement of conventional street lighting by LEDs produces complex and species-specific responses in bats.

## LEDs and Ecosystems

While experiments studying the impacts of ALAN on living organisms are on the increase, two aspects that need greater consideration have been only poorly investigated. One aspect is the impact on whole ecosystems, both aquatic and terrestrial. Indeed, species are linked by trophic or symbiotic interactions, and any type of impact of any anthropogenic pressure on one component of an ecosystem has consequences on the whole community, which may lead to remodelling or collapse of the entire system (Bennie et al., 2015a,b, 2016; Sanders et al., 2015; Zapata et al., 2019). Ascending and descending effects may be observed, depending on the trophic position of the species affected. Non-trophic interactions refer to the ALAN-induced impact on pollinating or seed dispersal species (more than 75% of global crops depend to varying degrees on animal pollination), or resource competition between species with diurnal, nocturnal or twilight activity and whose activity rhythms are altered by ALAN-induced photoperiod changes. The other aspect is the impact of concomitant or successive actions of a long list of anthropogenic factors, including physical (ALAN, noise, plastics…) and chemical (pesticides, herbicides, heavy metals, nanoparticles…) pollution, climate change (rise in temperatures, oceanic acidification, changing currents…), modification and reduction of natural spaces (urbanization, deforestation, and physical barriers), etc. Together they are likely to have more than additive effects, with severe implications on species and assemblages. These issues are discussed below.

### Aquatic Ecosystems: Grazing Fish and Sessile Invertebrates

Assessment of ALAN in coastal ecosystems, including estuaries, is limited (Zapata et al., 2019), although 60% of the world’s largest cities are located within 100 km of the coast, and more than 20% of coastal areas are exposed to ALAN (Bolton et al., 2017). A study conducted in Sydney Harbour (Australia) investigated the effects of ALAN using warm light LED spotlights that provided similar or lower levels of ALAN as recorded in other urban coastal cities. Under a natural LD cycle, fish abundance, all species combined, varied over the 24-h cycle (Figure 12A): overall, fish were more abundant, but more sedentary at night than during daytime, and predation on sessile invertebrates was higher during daytime (Bolton et al., 2017). ALAN modified this pattern with night predation increasing to levels observed during the day. Although the abundance of fish (including predators) was markedly reduced, predation on sessile invertebrates was increased. As a consequence, the structure of the sessile assemblage was disrupted at night, which may have dramatic consequences: these assemblages perform essential activities (spawning, settlement, and feeding) at night when predation pressure is low. The authors concluded that ALAN had implications for the structure of the trophic web system that might lead to altered functioning (Bolton et al., 2017). These data agree with investigations showing cool white LED lighting (19 lx or 30 lx at water surface) affected colonization by sessile and mobile benthic species (13 quantified), inducing reduction or suppression in some species while leading to increases in others (Davies, 2014). Imbalance of interspecific interactions were also shown from a study on Dog Whelks *Nucella lapillus*. *N. lapillus* are widely distributed across the North Atlantic (including illuminated coastal areas) and feed on barnacles and mussels; they were more likely to, respond to, and handle prey under, a white LED light (∼21 lx) compared to controls, irrespective of the presence of a snail predator (the common shore crab *Carcinus maenas*) (Underwood et al., 2017). Alterations of trophic interactions were also reported to occur under ALAN in studies performed along the Italian coast, where the population of grazing snails *Melarhaphe neritoides* has positive effects on the diversity of epilithic heterotrophic bacteria under a natural LD cycle (Maggi et al., 2020). ALAN (white LED, 27 lx) modified this by reducing the density of grazers (thus erasing the positive effects on heterotrophic bacteria) and increasing autotrophic Cyanobacteria. The authors concluded ALAN was likely to alter natural systems by annihilating positive interactions across trophic levels.

### Aquatic Ecosystems: Crossing Boundaries With Riparian Ecosystems

Artificial light at night, irrespective of the light source, induces redistribution of insect populations (Meyer and Sullivan, 2013; Davies et al., 2017), some species increase in number while others decrease. Globally, observations indicate significant alterations in the number of represented species and in the size and weight of individuals. For example, mimicking street lighting levels using wide spectrum LEDs at a few sites of the Ohio river (United States), resulted in a 44% decrease in the number of *tetragnathidae* spiders, a 16% decrease in biodiversity and a 76% decrease in the average body size of the species; conversely, the size of neighbouring terrestrial arthropods was increased by 309% (Meyer and Sullivan, 2013). The authors concluded ALAN altered the structure of communities in this system *via* changes in reciprocal aquatic–terrestrial fluxes of invertebrates. Another field study was conducted in the same area, studying the impact of ALAN (0 to 20 lx) provided by HPS and cool white LED lamps (Sullivan et al., 2019). At moderate to high levels of ALAN, the density of predatory orb-web spiders (*Tetragnathidae* and *Araneidae*) was particularly affected in riparian areas. At the community level, both density and family richness were affected, altering arthropod community structure; increasing ALAN induced larger proportions of predators wolf spiders, [*Lycosidae*]); rove beetles (*Staphylinidae*) and detritivores (*Oniscidae*), and smaller numbers of omnivores (ants [*Formicidae*]). In wetland systems, aquatic insect density increased and the composition of emergent insect families was different under LED or HPS or natural night lighting. ALAN also decreased the invertebrate food chain length and altered the flows of energy between aquatic and terrestrial systems (Sullivan et al., 2019).

### Terrestrial Ecosystems: Redistribution of Insect Populations

**FIGURE 13:**
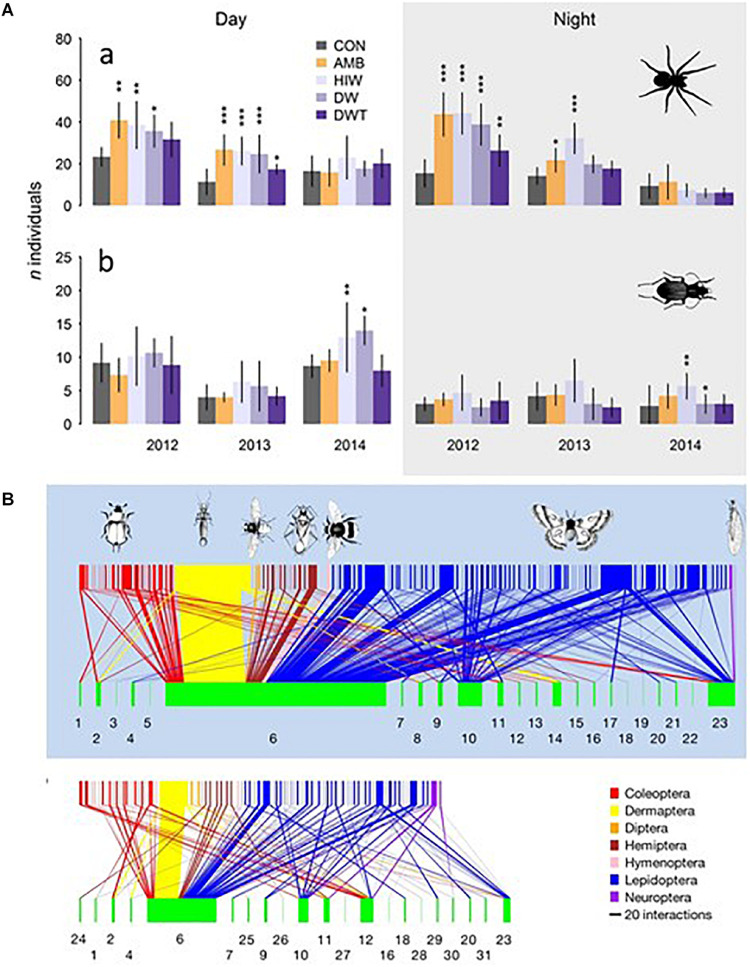
**(A)** The impact of alternative LED lighting strategies on the total numbers of individual grassland spiders (*Araneae*) (a) and beetles (*Coleoptera*) (b) caught in each year, respectively. LED lighting was equivalent to that experienced at ground level under LED street lighting for HIW (high-intensity white 29.6 ± 1.2 lx), under dimmed street lighting for DW (dimmed white, 14.6 ± 0.3 lx), or under timed dimmed street lighting for DWT (14.4 ± 0.8 lx, switched off between 00:00 and 04:00 GMT). AMB was amber lighting (18.2 ± 1.3 lx, λ_*max*_ = 588 nm). Controls (CON) experienced total darkness. Bar heights and error bars denote means 95% confidence intervals. Stars denote differences with the controls that were significant with 95% (*), 99% (**), and 99.9% or greater (***) confidence. From Davies et al. (2017). No special permission required. **(B)** Effects of artificial lighting on parameters of overall quantified nocturnal plant-flower visitor networks of seven dark sites (above) and seven experimentally illuminated sites (below). The rectangles represent insect species (top) and plant species (bottom), and the connecting lines represent interactions among species. Species codes for the plants and a list of insect species are given in Knop et al. (2017). The study was run in 14 sites of the Swiss Alps; illumination was using neutral white LED street lamps (4,000K) that provided 52.0 ± 4.2 lx on the ground. Adapted from Knop et al. (2017). More details in the original publication. With permission.

Attraction by light and redistribution of populations characterize the effects of ALAN on insects. Attraction of flying insects is a well-known phenomenon. At Hawkes Bay (New Zealand) the number of flying insects captured under street lighting sources was 48% higher when using white LED (2700 to 6500 K) than with HPS lamps (Pawson and Bader, 2014). The authors suggested that the replacement of sodium lamps by LEDs is likely to increase the deleterious effects of ALAN with unpredictable consequences, as harmful species (*e.g*., the patchy Bombyx *Lymantria dispar* whose caterpillars attack forests) might develop at the expense of endemic species. Also, most moths attracted by street lamps abandon fields and open ground, leading to population decreases of 50%, while biodiversity is also reduced by ∼25% (Macgregor et al., 2015, 2017). Redistribution of surface terrestrial insect communities has been observed near light sources, regardless of the time of day or night (Davies et al., 2012, 2017; Bennie et al., 2015a). In a 3-year experiment, it was shown that nocturnal lighting by LEDs of different quality and intensity altered the distribution of arachnid and coleopteran species on the ground (Davies et al., 2017; Figure 13A). The effects were diminished, but not suppressed, upon reduction of intensity or duration of the light signal. Species of predators and scavengers were most represented near lighted areas, suggesting an alteration of the local ecosystem.

Street lighting also increased the activity of flying insects in the surroundings. They are thus likely to carry less pollen, with possible consequences on plant pollination. Such a phenomenon has been observed in a field study at sites that had never previously experienced ALAN and carried an identical variety of plants (*Cirsium oleraceum, Eupatorium cannabinum, Valeriana officinalis, Epilobium angustifolium, and Silene vulgaris*) (Knop et al., 2017). Half of the sites were illuminated at night using white LEDs (4000 K), the other half remained in the dark. Under these conditions ALAN reduced visits of pollinating nocturnal insects by 62%, with negative consequences on the reproduction of plants. In addition, the diurnal population of pollinating species was also negatively impacted. The result was a general reduction of plants as well as the insects that feed on them (Figure 13B; Knop et al., 2017). Direct and indirect effects have also been observed in a field study in the Denver area (CO, United States), investigating the impact of ALAN (HPS lamps) on the relationship between the Smooth Brome *Bromus inermis* and larvae of the moth *Apamea sordens* that feeds on seed heads and leaves (Grenis and Murphy, 2019). Plants growing under normal periodic darkness were hardier than those under street lamps, and the effects of street lighting on larvae were both direct (larvae were smaller when reared under streetlights) and indirect (plant traits led to reduced larval growth).

### Terrestrial Ecosystems: Plants, Insects and Their Parasites

Sanders and colleagues investigated the impact of white LED street lighting (30 lx) in a plant-aphid-parasitoid community. The first investigation included three aphid species, *Aphis fabae, Acyrthosiphon pisum* and *Megoura viciae*; their parasites, respectively *Lysiphlebus fabarum, Aphidius ervi*, and *A. megourae*; and the aphids’ food source, the broad bean *Vicia faba* (Sanders et al., 2015, 2018). In the absence of anthropogenic pressure this community is very stable. ALAN reduced bean plant biomass and, most likely as a result of bottom-up effects, the abundance of two aphid species by 20% over five generations. For *M. viciae* the effect was reversed under autumnal conditions (ALAN promoting continuous reproduction of the species). All three parasitic species were negatively affected by ALAN, as a result of host number reduction (Sanders et al., 2015). The second investigation (greenhouse and field experiments) tested the effects of different illuminance levels (0.1 to 100 lx) on the same mesocosm that also included barley *Hordeum vulgare*, as a resource for the aphid *Sitobion avenae*, and *Praon dorsale*, which attacks the three aphids *S. avenae*, *A. pisum* and *M. viciae*. The lowest levels of ALAN (0.1 to 5 lx; equivalent to severe sky glow) induced the strongest effects, reducing aphid densities by 45% as a result of parasite being twice more efficient in attacking aphids. The effects were reversed at higher light intensities because the parasites spent less time on their hosts (Sanders et al., 2018). *M. viciae* was the main aphid species affected, while *A. fabae* responded with a negative effect at 10 lx and a positive effect at lower or higher intensities; *S. avenae* was not affected. There was a positive relationship between plant biomass and light intensity in the greenhouse experiment, while in the field only *V. faba* responded (and only at 20 lx illuminance level). According to the authors, while not discarding a possible bottom-up effect through increased plant biomass (providing more resources for aphids under higher light intensities), the interaction between aphids and parasites was the critical driver for the responses observed in the field experiment (Sanders et al., 2018).

### Terrestrial Ecosystems: Bats, Moths, and Pollination

**FIGURE 14:**
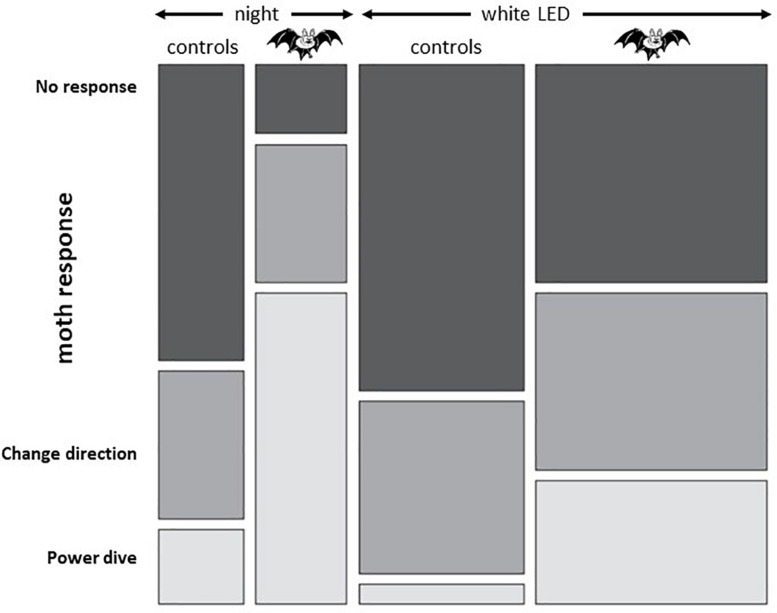
The mosaic plot illustrates the proportion of moth flight responses under four different conditions: absence or presence of bats (*Nyctalus* sp.) under total darkness or white LED illumination, in the area of Bristol (United Kingdom). Moths respond to the presence of bats under unlit conditions at night by escape movements. This escape behaviour is markedly affected in the presence of white LED. Column width is proportional to sample size. From Wakefield et al. (2015). No special permission required.

The impacts on bats (as reported in “Animals”) have major consequences on insect populations, especially moths (Minnaar et al., 2015; Wakefield et al., 2015). The attraction that ALAN exerts on insects in general, and moths in particular, is one reason why their world population is steadily decreasing (Macgregor et al., 2015, 2017). Attraction of moths by ALAN induces alterations in behaviour (flight, foraging or searching for sexual partners) and reproductive function. In addition, ALAN also disturbs the ultrasound detection system that some moths (*Geometridae, Noctuidae*, or *Notodontidae*) use to detect bat predators (Figure 14; Wakefield et al., 2015). A major consequence is the widespread reduction in moth populations and a redistribution of insect populations in the local environment. Remodelling of this kind is likely to have consequences for the entire ecosystem, affecting both plants (because moths are among the largest pollinators across the globe; see section “Aquatic Ecosystems: Crossing Boundaries With Riparian Ecosystems**”** above) (Macgregor et al., 2015, 2017, 2019), and other predators (spiders and small vertebrates) that feed on these moths. Consequently, ALAN constitutes a short-term advantage for flying predators, while disadvantages appear in the medium- and long-term, with the risk of increased bat mortality (due to collision) and the scarcity of prey leading to negative population dynamics (Altringham and Kerth, 2015; Azam, 2016).

The survival of some plants is also likely to be affected by decreases of fruit-eating and nectar-eating bats (Lewanzik and Voigt, 2014). *Carollia sowelli* is an American tropical bat species important in seed dispersal of *Piperaceae* (pepper) and *Solanaceae* (potato, tomato, eggplant, chili pepper). Bats are repelled by light: in the presence of 4.5 lx HPS lighting their activity was reduced by 50%, fruit consumption by 20% and the hour of consumption delayed by more than 100%. Accordingly, this may have harmful consequences on plant reproduction (Macgregor et al., 2015, 2017). The authors concluded that more studies are needed to further elucidate the impact of ALAN on bats and the plants that rely on them for seed dispersal and pollination (including plants of agricultural importance such as tea).

## LEDs and Other Anthropogenic Factors: Some Examples

The continuous increase of human activities leads to permanent reorganization of spaces. The extension of urban and peri-urban areas, industrial and agricultural surfaces, communication routes (roads, railways, sea lanes), all lead to decreases in, and fragmentation of, natural habitats. With this come additional threats: obstacles (dams, pumps, and turbines), physical pollutants (light, noise, plastics and other trash), chemical pollutants (including endocrine disruptors [polychlorobiphenyls (PCBs), synthetic steroids, organochlorine pesticides, detergents, etc.], nanoparticles, heavy metals, radioactive waste…) and climate change (rising temperatures, ocean acidification …). Thus, artificial light either during daytime or night-time, is not the only anthropogenic pressure on wildlife, and the question arises as to what is the impact of simultaneous and/or successive actions of these factors, since many of these targeting the same organs or associated and interconnected organs as is the case for the neuroendocrine system of vertebrates (Figure 11). In more than half the cases, simultaneous action of several of these factors resulted in synergistic or cooperative effects, while in other cases the effects were additive or even antagonistic (Mora et al., 2007; Darling and Côté, 2008; Côté et al., 2016). For example, overexploitation, temperature rise or habitat fragmentation, taken independently, all induce a decline in rotifer population; but taken together the rate of decline is increased by 50-fold (Mora et al., 2007). The number of studies reporting on the combined effects of ALAN and other anthropogenic factors remains scarce.

### Frogs and Midges

The singing behaviour of the male frog *Engystomops pustulosus* is intended to attract females at night. A parasite of *E. pustulosus*, the fly *Corethrella* spp., is also only attracted by the song of the male at night, as during the day they are eaten by the host. In urban areas, both noise and light affected the singing behaviour of the male; and both, noise (by acoustic interference) and light (by reducing locomotor activity) diminished the ability of the parasitic midge to locate and feed on its host (McMahon et al., 2017). The combination of the two anthropogenic factors was dramatic as it led to total disappearance of the midges. The authors highlight the need to consider the multiplicity of urban anthropogenic factors in community impact studies.

### Birds and Noise

The great tit *Parus major* is a diurnal species very sensitive to ALAN (see section “Birds”). Under a natural LD cycle tits display rhythmic diurnal activity patterns, which differ slightly between urban and forest birds (Dominoni et al., 2020). Both ALAN and noise affect this pattern in opposite ways: ALAN increased the overall activity while noise had the opposite effect. Both factors together had synergistic effects on night-time activities, but the effects were antagonistic for daytime activity. Moreover a significant difference was found between urban and forest birds as the interactive effects of light and noise on daytime, night-time, dusk-time and offset of activity were seen in urban but not forest birds (Dominoni et al., 2020).

### Bats and Roads

Roads destroy, fragment and reduce surface habitat, degrading habitat by introducing physical barriers, noise, light and chemical pollution, and inducing lethal injuries through collision with traffic. The effects on avian and mammalian populations (in decline) can be seen up to several km away from the roads. Bats are particularly affected by all the above-mentioned factors in a species-dependent manner (Altringham and Kerth, 2015). For example, populations of small and low-flying bats are more affected than those of large high-flying bats. Most importantly, the above-mentioned factors exert cumulative effects with dramatic consequences that may only appear after several generations (Altringham and Kerth, 2015).

### The Migrating Atlantic Salmon - A Case Study

**FIGURE 15:**
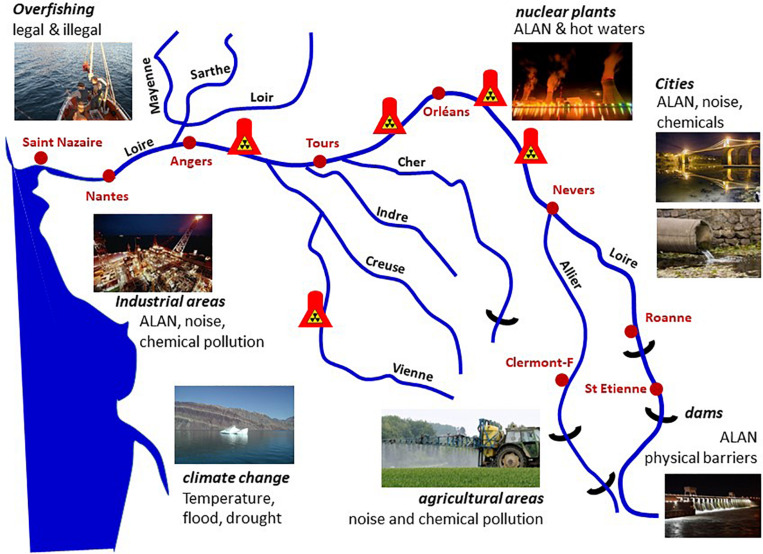
Migration is a crucial event in the Atlantic salmon, *Salmo salar*. In the Loire/Allier basin a ∼800 km downstream migration brings young smolts from their hatching area to the sea, where they feed and mature. In the journey they have to face light pollution (ALAN) when crossing cities or areas of active human activities (nuclear plants, industrial areas, harbours) as well as a series of other threats of anthropogenic origin, including physical barriers, overfishing, water temperature rise, physical (noise) and chemical (*e.g*., endocrine disruptors) pollution. They must run another 800 km back when returning to the spawning grounds. Altogether this addition of threats impacts on metabolic reactions and physiological regulation, including their rhythmic components, which have put the species in danger of extinction.

Catches of Atlantic salmon, *Salmo salar*, from the Loire/Allier (France) basin have dropped from 30,000 at the end of the 19th century to less than 1500 nowadays (Marchand et al., 2017), without mentioning a dramatic reduction in the size of the captured animals. This population decay is due to a chain of cascading reactions (Figure 15): (i) natural predation; (ii) overfishing (recreational, industrial, and poaching), (iii) sporadic and continuous chemical pollution due to urban and agricultural activities (including endocrine and metabolic disruptors), (iv) physical pollution due to ALAN, which affects vision (because of the strongly illuminated bridges and buildings; see section “Fish”), rhythmic metabolism, and behaviour (locomotor activity, daily vertical migration as well as down-stream and upstream migration), noise and temperature (due to global warming as well as release of warm waters from nuclear run-off basins), (v) physical barriers (pumps, turbines [particularly from nuclear plants], dams and control of water flows) (Figures 11, 15). These are multiple sources of nuisance affecting metabolism, physiology and behaviour (Scholz and Mayer, 2008; Casals-Casas and Desvergne, 2011; Lambert et al., 2015; Bedrosian et al., 2016). Salmon navigating long distance rivers are likely to be more affected than others, as they will face a concomitance and/or succession of these factors along a course of at least 700 km. Laboratory investigations have provided evidence that the effects of combining LED lights with endocrine disruptors or temperature changes depend on the wavelength (Figure 11). In the perciform *Oplegnathus fasciatus*, bisphenol A activated hepatic and plasma markers of oxidative and lipid stress, increased DNA degradation and cell apoptosis and decreased melatonin and circulating immunoglobulins; these effects were mitigated by green (530 nm) but not red (620 nm) LED light of 0.3 and 0.5 W/m^2^ (Choi et al., 2016). Similar results were obtained in *C. auratus* (Jung M. M. et al., 2016). In addition, in the latter a rise in temperature of 22 to 30°C induced (1) an increase in glucose, cortisol, T3 and T4 thyroid hormones in the blood and (2) T3 and T4 receptors in the brain, but (3) a decrease in hepatic and plasma immunoglobulins. Green LED lighting or the administration of melatonin, reversed these effects (Jung S. J. et al., 2016). Figure 11 provides a schematic presentation of how many of these factors are likely to affect the fish neuroendocrine system.

## Conclusion

Recent years have seen a growing global awareness of the potential negative consequences of exposure to ALAN. Over the last 20 years both the number of light-emitting sources, and the intensity of radiated light, have increased dramatically across the surface of the globe, not only within vast tracts of urbanized land but also along coastal areas and even in relatively isolated regions like deserts, mountain ranges and open ocean. A fierce debate has arisen in many countries as documented scientific evidence has begun to suggest that prolonged exposure to ALAN can have adverse effects on human health, with a substantial number of studies indicating links between ALAN and sleep loss and fatigue on the short term, and cancer, metabolic syndrome, mental health and cognitive disturbances on the long term (Lunn et al., 2017). Much of the scientific rationale underpinning these effects concerns the disruptive effects of ALAN upon the proper synchronization of the circadian clock, a fundamental regulatory system, which exists in virtually all living organisms and originated at the beginning of evolution. The over-riding principle of circadian networks is that they align inner physiology with the natural day-night cycle, in order to optimize energy expenditure. It is hence obvious that exposure to ALAN creates a temporal disturbance leading to misalignment of physiology and metabolism with the fluctuating day-night cycle. The paramount importance of this system is now recognized in subjects as diverse as agriculture and medicine, and was recently highlighted by the attribution the 2018 Nobel Prize in Medicine and Physiology to the three pioneers in the field of chronobiology.

In vertebrates including humans, a key clock-mediated process involves altered secretion of melatonin, a neurohormone involved in the regulation of many rhythmic processes but also as promoting antioxidant protection in the brain and elsewhere. Melatonin has strong impact on the neuroendocrine system. Normally secreted only during the dark, nocturnal light exposure diminishes or even suppresses melatonin secretion, which if occurring over a long period leads to overall deprivation in melatonin, with consequent problems (*e.g*., sleep) and potentially longer-term effects (*e.g*., on cognition, metabolism (diabetes), fertility and heart disease). It has been argued that ALAN can be considered as a source of endocrine disruption in human, since so many hormones, pheromones and metabolites are under circadian control (Russart and Nelson, 2018). This is strengthen by the observation that ALAN together with other external cues and disruptors often target the same neuroendocrine areas in vertebrates (Figure 11).

While the scientific literature is beginning to report many studies showing possible detrimental side-effects of ALAN upon human health and well-being, the effects of ALAN on the natural world, both flora and fauna, has been less talked about and is less prominent in the public consciousness. The constant increase in ALAN through anthropogenic activity means that nowadays large areas of the earth’s surface (even including oceans) are permanently bathed in light, obscuring the natural order of alternating periods of light and darkness. The day-night cycle, and also that of seasonal changes, is a critical aspect of the adaptive responses of living organisms to their shifting environment, and a correct « reading » of these cycles is essential to the correct timing of such processes as flowering, reproduction and foraging, among many others. Living organisms have developed a huge variety of strategies to integrate the visual information and to decode time. It is not surprising therefore that ALAN impacts natural systems at all levels of organization, from unicellular to eukaryotes, from systems physiology to community structures, from population behaviour to trophic interactions.

The mechanisms of light capture and of adaptation to the daily and annual changes in photoperiod started at the origin of life, and have become increasingly complex over billions of years of evolution. ALAN is now challenging this in a time scale of decades only. The ongoing extension of urban areas contributes to the cumulative effects of ALAN together with a range of anthropogenic pressures on wildlife and ecosystems (demography, over-exploitation of resources, physical obstacles, reduction of natural spaces, pollution, climate change, etc.). The result is a dramatic acceleration in extinction of species, followed by disorganization and collapse of ecosystems. The great majority of species is unable to overcome such additive stress factors and to develop new strategies in such a short period of time. Reversing or even slowing down this process will need a profound reconsideration of our environmental policies, which implies re-examination of our modern life style. With regard to ALAN the international political decision to replace pre-existing lighting systems with LED may further complicate the current scenario, due primarily to a wider emission spectrum and an enriched emission of short wavelength light to which circadian clocks are particularly sensitive. We propose that efforts should be made to limit night-time illumination to more essential purposes (*e.g*., road safety), within more narrowly defined areas and at more restricted hours. The use of directed lighting to minimize wasted un-useful radiation and with carefully selected spectral emissions should permit human activity to continue unhindered while significantly reducing the impact on species.

“*What we conserve defines what we are or pretend to be. We must establish and promote comprehensive dialogs among social scientists, ecologists, and evolutionary biologists to explore the biological and cultural roots of our interactions with nonhumans and to understand the origins of our inertia in the face of the urgency of biodiversity erosion. Addressing this major challenge for humanity may also enhance our ability to respect each other in our societies*” (Sarrazin and Lecomte, 2016).

## Author Contributions

JF coordinated the work and wrote the manuscript. AT, CG, CM, DA, FB-C, FV, and DH contributed to discussions and reading of the manuscript. DH contributed to English editing. All authors contributed to the article and approved the submitted version.

## Conflict of Interest

The authors declare that the research was conducted in the absence of any commercial or financial relationships that could be construed as a potential conflict of interest. The handling editor declared a shared affiliation with one of the authors DH at the time of review.

## Footnotes

1 which use light as a source of energy and CO_2_ as a source of carbon.

## Abbreviations

ALAN: Artificial Light at Night
ccg: clock-controlled gene
Cry: cryptochromes
D: darkness
DD: constant dark
FSH: folliculo-stimulating hormone
HPS: high pressure sodium
ipRGCs: intrinsically photosensitive retinal ganglion cells
JH: juvenile hormone
L: light
LD: alternation of light and darkness
LH: luteinizing hormone
LL: constant light
LED: light-emitting diode
LOV, light: oxygen or voltage
LPS: low-pressure sodium-vapour
LWS: long wavelength sensitive opsin
PCB: polychlorobiphenyl
PDF: pigment-dispersing factor
Rh: rhodopsin
SWS1: short wavelength sensitive opsin
THS: thyroid stimulating hormone.
